# Upregulation of IGF2R evades lysosomal dysfunction-induced apoptosis of cervical cancer cells via transport of cathepsins

**DOI:** 10.1038/s41419-019-2117-9

**Published:** 2019-11-20

**Authors:** Takashi Takeda, Masayuki Komatsu, Fumiko Chiwaki, Rie Komatsuzaki, Kanako Nakamura, Kosuke Tsuji, Yusuke Kobayashi, Eiichiro Tominaga, Masaya Ono, Kouji Banno, Daisuke Aoki, Hiroki Sasaki

**Affiliations:** 1 Department of Translational Oncology, National Cancer Center Research Institute, Tokyo, 104-0045 Japan; 2 Department of Obstetrics and Gynecology, Keio University School of Medicine, Tokyo, 160-8582 Japan; 3 Department of Clinical Proteomics, National Cancer Center Research Institute, Tokyo, 104-0045 Japan

**Keywords:** Targeted therapies, Cervical cancer, Apoptosis

## Abstract

Cervical cancer is the most common gynecological malignancy in the world; however, the survival rates of advanced-stage and recurrent cervical cancer patients remain poor. The multifaced protein insulin-like growth factor 2 receptor (IGF2R) has various ligands, represented as IGF-2 and mannose-6-phosphate (M6P)-tagged proteins. Regarding its antagonistic activity as an IGF1R signal, *IGF2R* is currently considered a tumor suppressor gene, whereas its significance as an M6P receptor is still unclear. Here, on the basis of transcriptome analysis of TCGA and GEO open datasets, we show that IGF2R is upregulated and correlated with poor prognosis in cervical cancer. Several experiments using cervical cancer cell lines revealed that IGF2R depletion induced apoptosis, decreased cell viability, and increased vulnerability to certain anticancer drug cisplatin. In contrast to its negligible impact in IGF1R signaling, loss of IGF2R disrupted the Golgi-to-lysosome transport of M6P-tagged cathepsins, resulting in decreased lysosomal activity, with their abnormal accumulation and dysfunction of both autophagy and mitophagy, which cause the accumulation of misfolded proteins and production of reactive oxygen species. Taken together, IGF2R has an oncogenic role through transportation of M6P-tagged cargo in cervical cancer and can be used as a predictive biomarker for prognostic classification.

## Introduction

Cervical cancer is the most common gynecological malignancy in the world. Approximately 311,000 of affected patients died in 2018, accounting for 7.5% of cancer-related deaths worldwide^1^. Cytology screening and HPV vaccines constitute a successful prevention program in some developed countries, but the coverage is still low globally^2,3^. Currently recommended treatment options for patients with early-stage cervical cancer are surgery or radiotherapy, both of which achieve good clinical outcomes globally^3^. Radiotherapies with/without cisplatin-based concurrent chemotherapies are established as the standard care for patients with advanced-stage cervical cancer, although 5-year survival rates remain poor^3^. Thus, discovering new modalities for the treatment of patients through the molecular understanding of their malignancies and treatment resistances is of utmost exigency.

The cation-independent mannose 6-phosphate/insulin-like growth factor 2 receptor (CI-M6P/IGF2R, hereafter IGF2R) is a type-1 transmembrane glycoprotein consisting of a large N-terminal extracytoplasmic domain, which allows it to bind to a wide variety of ligands^4–7^. It has two major types of ligands: insulin-like growth factor 2 (IGF-2) and mannose 6-phosphate (M6P)-labeled glycosylated proteins, both of which have distinct but important roles in normal development and homeostasis in mammals. Many studies support the idea that IGF2R on the plasma membrane indirectly suppresses insulin-like growth factor 1 receptor (IGF1R) signaling by scavenging extracellular IGF-2^8^. Furthermore, several lines of evidence demonstrate that IGF2R binds to M6P-labeled proteins to transport de novo synthesized proteins to lysosomes. In some cancers, high frequencies of loss-of-heterozygosity (LOH) at the IGF2R locus have been reported^8–11^. Furthermore, in some cancers, the ectopic expression of IGF2R suppresses tumor growth^12,13^, whereas knockdown of the receptor results in the opposite phenotype^14,15^. Therefore, IGF2R is currently considered a tumor suppressor gene. However, the ligands of the receptor critically contributing to the suppression of tumors remain to be identified.

Here, we identified IGF2R as a poor prognostic biomarker for cervical cancer patients and showed that it has oncogenic functions, contrary to previous reports about other cancers. IGF2R knockdown causes insufficient transport of lysosomal enzymes to lysosomes, which in turn triggers the accumulation of aberrant lysosomes with low degradative activities. Consequently, the loss of autophagic and mitophagic functions induces the accumulation of harmful reactive oxygen species (ROS), and aggregated proteins in the cells cause apoptosis. Similar effects can be observed by the inhibition of casein kinase 2 (CK2) which has been reported to phosphorylate cytoplasmic tail of IGF2R that is necessary for the Golgi-to-lysosome transportation function. To our knowledge, this is the first report of IGF2R as a therapeutic target, which may lead to further development of both diagnostic and treatment modalities.

## Results

### IGF2R is a poor prognostic biomarker in patients with cervical cancer

**Fig. 1:**
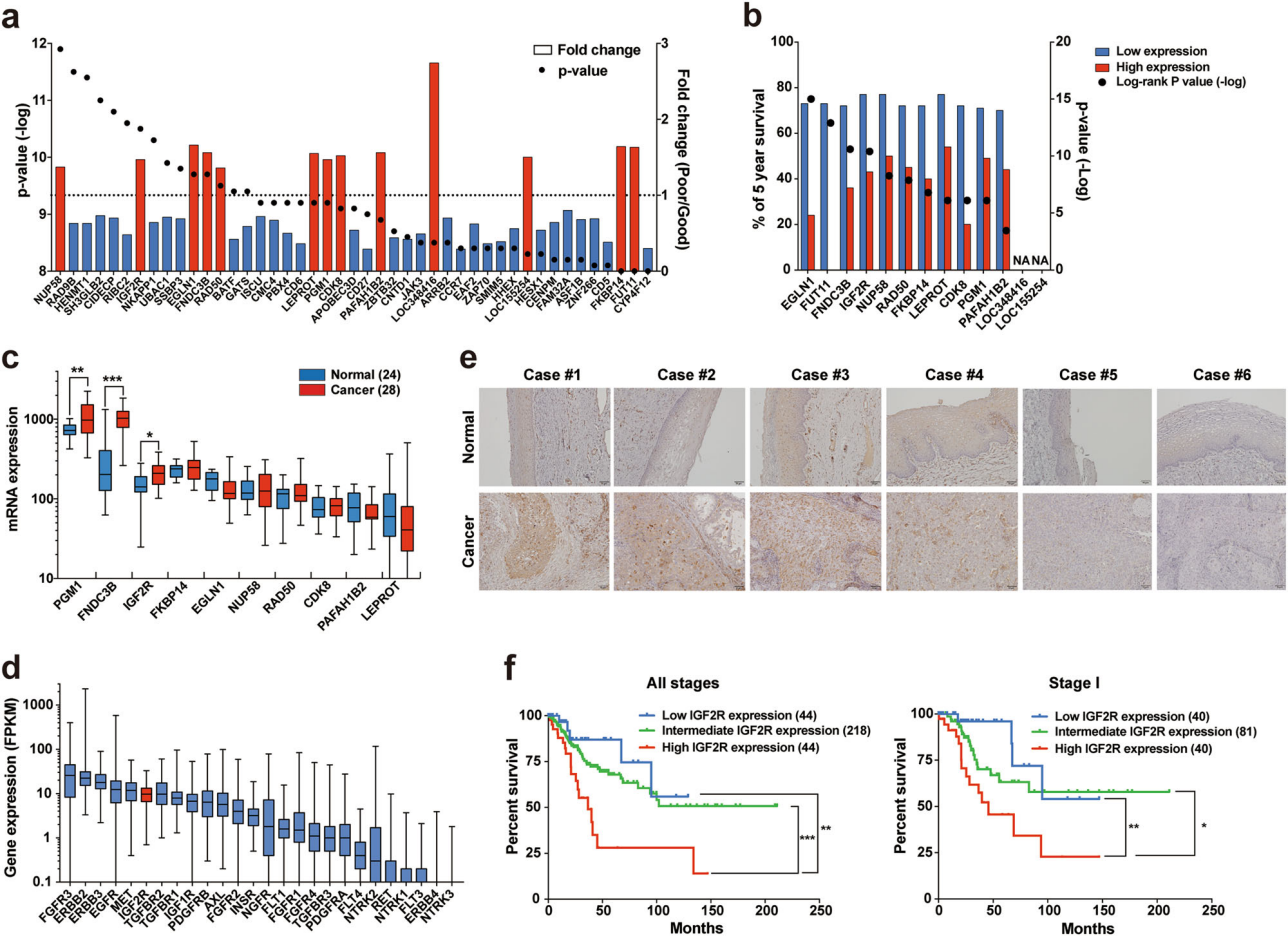
Aberrant expression of IGF2R is a poor prognostic factor in patients with cervical cancer. **a** Identification of genes with decreased or increased expression in patients with poor prognoses. Each dot and bar indicate the *p*-value and fold change, respectively. Fold change values were calculated by the average expression in short-term survivors (died within 2 years after diagnosis; 43 cases) divided by that of long-term survivors (alive over 4 years after diagnosis; 52 cases). Red and blue bars indicate high and low expression in short-term survivors compared to long-term survivors, respectively. Candidate genes are arranged in ascending order. **b** Correlation between patient survival and the mRNA expression levels of genes identified as having increased expression in short-term survivors. Each dot indicates the log-rank *p*-value. Blue and red bars indicate the percentage of 5-year overall survival in patient groups with low and high expression levels, respectively, of the corresponding genes. NA, data not available. **c** Differential gene expression between normal cervix (24 cases, blue box) and cervical cancer (28 cases, red box) samples. **d** Gene expression of major receptor tyrosine kinases. Each mRNA expression level is normalized to the FPKM value. Boxes and bars represented the range from first to third quartile and minimum to maximum, respectively. **e** Immunohistochemistry of IGF2R in normal cervix and cervical cancer tissues. Scale bar, 50 μm. **f** Overall survival analyses of cervical cancer patient groups classified by IGF2R mRNA expression. *, **, and *** represent *p* < 0.05, *p* < 0.01, and *p* < 0.001, respectively.

Thirteen candidate genes were identified as being highly expressed in poor survivors vis-à-vis good survivors (Fig. 1a). Overall survival analysis confirmed that 11 candidate genes out of the 13 were independent poor prognostic factors in cervical cancer (Fig. 1b). A publicly available DNA microarray dataset revealed that three of the candidates (*PGM1*, *FNDC3B*, and *IGF2R*) were aberrantly expressed in cervical cancer tissues relative to normal cervix tissues (Fig. 1c). Finally, we focused on the gene *IGF2R* because its mRNA expression is higher than that of other oncogenic receptors in cervical cancer tissues (Fig. 1d). Consistent with the DNA microarray analysis results, immunohistochemical staining showed higher IGF2R expression in cervical cancer tissues (four cases out of six), whereas only weak staining was observed in their corresponding normal cervical tissues (Fig. 1e). A multi-omics analysis revealed that genetic alterations in IGF2R tended to be mutually exclusive of those in IGF1R but not of those in either insulin receptor (INSR) or their ligands (Supplementary Fig. S1a). However, correlation analysis showed no relationship among their mRNA expression levels (Supplementary Fig. S1b). To achieve a meaningful overall result from the analyses of these receptors, patients were classified into three groups based on their median mRNA expression level of each individual gene and its standard deviation (Supplementary Fig. S1c). Patients with high IGF2R expression showed significantly worse cervical cancer prognosis (Fig. 1f). In contrast, no such tendency was observed in IGF1R or INSR (Supplementary Fig. S1d). It is noteworthy that high IGF2R expression was also unfavorable for patients with stage I cervical cancer (Fig. 1f), indicating its clinical utility as a prognostic marker during early diagnosis. Overall survival analyses also revealed that high IGF2R expression is a poor prognostic factor not only for cervical cancer but also for breast and ovarian cancers. Furthermore, high expression of IGF2R was correlated with good prognosis in renal cancer and melanoma; however, for the latter, this was not significant (log-rank test; Supplementary Fig. S1e). In cervical cancer, IGF2R expression was correlated with clinical staging but not with distal metastasis or primary therapy outcomes (Table 1). In fact, there was no change in IGF2R mRNA expression before and after therapy (Supplementary Fig. S1f). Considering that progression-free survival was shorter in patients with high IGF2R expression (not shown), the receptor may play a role in recurrence in cervical cancer patients.

**Table 1:** Relationship between IGF2R expression and clinicopathological factors in patients with cervical cancer.

Variables	Number of cases	*IGF2R* expression	*P*-value
Age (years)			
<50	182	2,739	0.209
≥50	123	2,950	
Histological type 1			
Squamous cell carcinoma	258	2,834	0.777
Adenocarcinoma	47	2,768	
Histological type 2			
Keratinizing	55	3,086	0.14
Non-keratinizing	119	2,756	
Lymph node status			
No metastasis	130	2,724	0.716
Metastasis	71	2,652	
Distal metastasis			
No metastasis	135	2,657	0.202
Metastasis	21	3,190	
Primary therapy outcome			
CR/PR	141	2,947	0.551
SD/PD	24	3,149	
TMN staging			
Stage I	161	2,678	0.032
Stage II–IV	137	3,027	
TMN staging			
Stage I	161	2,678	-
Stage II	69	2,991	
Stage III	46	2,854	
Stage IV	22	3,501	

### Loss of IGF2R induces apoptosis and drug sensitivity in cervical cancer cells

**Fig. 2:**
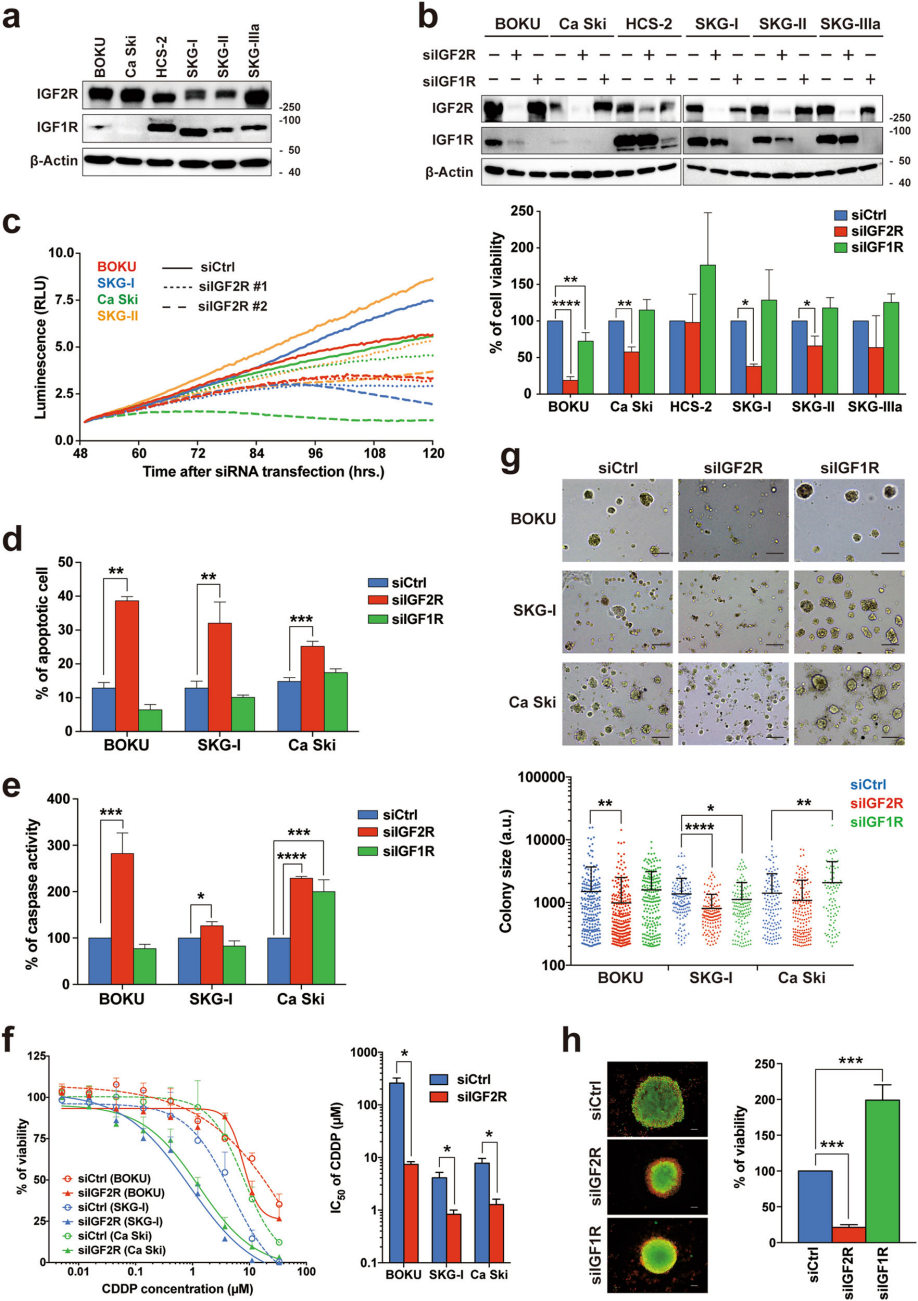
Knockdown of IGF2R induces apoptosis and drug sensitivity in cervical cancer cells. **a** Western blot analysis of IGF2R and IGF1R in cervical cancer cell lines. **b** Transient knockdown of IGF2R and IGF1R in cervical cancer cells. At 144 h after siRNA transfection, both the knockdown levels of the genes (top panel) and cell viability (bottom panel) were analyzed by western blot analysis and cell viability assay, respectively. **c** Time course analyses of cell growth under IGF2R knockdown (siIGF2R#1: s7219 and siIGF2R#2: HSS105256). **d** Apoptosis status of cells with IGF2R or IGF1R knockdown. Apoptotic cell populations (Annexin V-FITC^+^) at 144 h after siRNA transfection were analyzed by flow cytometry. Dot plots are shown in Supplementary Fig. S2c. **e** Total activities of caspase 3 and 7 of IGF2R- or IGF1R-knockdown cells at 144 h after siRNA transfection. **f** Sensitivity of IGF2R-knockdown cells to cisplatin. At 48 h after siRNA transfection, cells were further exposed to various concentrations of cisplatin for 96 h. The left and right panels show the dose-response curves and IC_50_ values for cisplatin (CDDP). **g** Matrigel-based three-dimensional culture assay at 12 days after siRNA transfection. Representative images of colonies and colony size are shown in the top and bottom panel, respectively. a.u.: arbitrary unit. **h** Spheroid-forming abilities of cells cultured on a non-adherent plate at 12 days after siRNA transfection are detected by live and dead staining (left panel). Green and red staining indicates viable and dead cells, respectively. The cell viability of the cultured spheroids was validated by monitoring their ATP levels (right panel). All the error bars represent the standard deviation of three independent experiments. *p *<* 0.05, **p *<* 0.01, ***p *<* 0.001, ****p *<* 0.0001; *t*-test with Welch’s correction and Dunnett’s multiple comparisons test. Scale bars represent 100 μm.

To investigate the biological significance of IGF2R and IGF1R in cervical cancer cells, we analyzed six cervical cancer cell lines using gene knockdown. In each gene, the mRNA expression levels correlated positively with the protein expression levels (Supplementary Fig. S2a). Consistent with a correlation analysis of an RNAseq dataset of cervical cancer tissues (Supplementary Fig. S1b), there was no relationship between IGF2R and IGF1R protein expression (Fig. 2a). Although IGF2R knockdown significantly decreased the cell viability of four cell lines out of six with time, no positive or negative impact on cell viability was observed after IGF1R knockdown (Fig. 2b, bottom panel, and Fig. 2c). Although neither IGF2R nor IGF1R knockdown altered cell cycle status in cervical cancer cells (Supplementary Fig. S2b), only apoptotic cells and caspase activities were significantly increased by IGF2R knockdown (Fig. 2d, e, and Supplementary Fig. S2c). Interestingly, IGF2R knockdown also enhanced cell sensitivity to cisplatin (Fig. 2f), suggesting that IGF2R contributes to chemotherapy refractoriness. Conversely, the knockdown effects on both cell migration and invasion were negligible (Supplementary Fig. S2d, e). Finally, IGF2R knockdown decreased both colony- and spheroid-formation abilities (Fig. 2g, h). IGF1R seemed to be affected by IGF2R knockdown, especially in the BOKU cell line. Western blotting with several IGF2R siRNAs showed that IGF1R expression was not affected in most cases of IGF2R knockdown transfection (Supplementary Fig. S2f). We selected siIGF2R#2, in which IGF1R expression was not decreased, and validated the cell viability (Supplementary Fig. S2f and Fig. 2c). IGF2R knockdown by siIGF2R#2 also decreased colony-formation ability and enhanced cell sensitivity to cisplatin in BOKU cells (Supplementary Fig. S2g, h). Thus, the loss of IGF2R induces caspase-dependent apoptosis and drug sensitivity in cervical cancer cells.

### The oncogenic functions of IGF2R are independent of IGF1R signaling

**Fig. 3:**
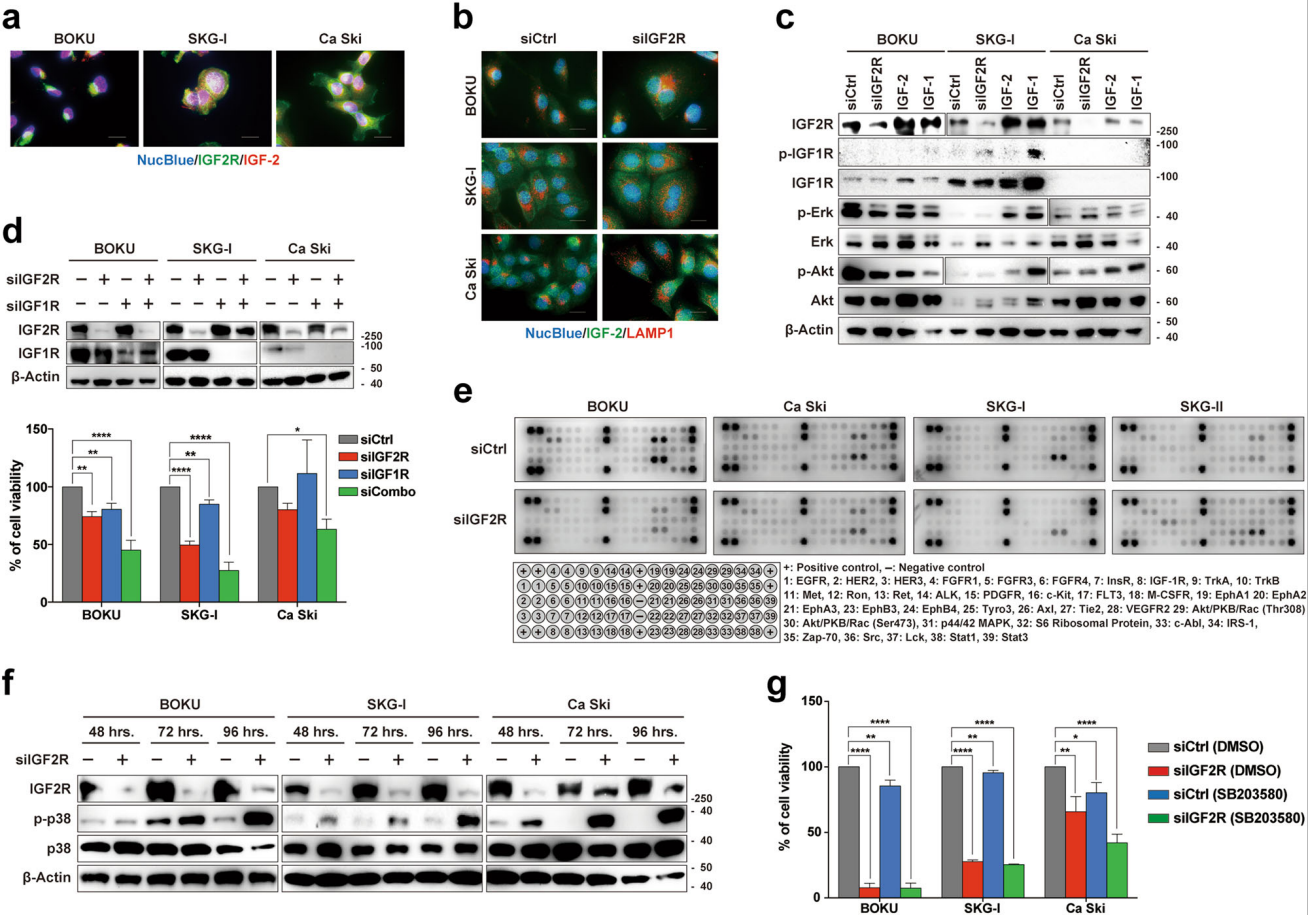
The neutralization effect of IGF2R on IGF1R signaling is negligible in cervical cancer. **a** Co-localization of IGF2R (green) and IGF-2 (red) in cervical cancer cells. **b** Influence of IGF2R on IGF-2 degradation. At 96 h after IGF2R knockdown, cells were stained with anti-IGF-2 (green) and anti-LAMP1 (red) antibodies. **c** Activation of IGF1R signaling pathways in cervical cancer cells. The phosphorylation of IGF1R and its downstream kinases were analyzed by western blot analysis. In addition to IGF2R knockdown samples at 24 h after siRNA transfection, other samples that recovered at 15 min after stimulation by their ligands (IGF-1 and IGF-2) were also analyzed. **d** Combined effect of IGF2R and IGF1R knockdown on cell viability. The knockdown of each receptor was confirmed by western blot analysis (top panel). The bottom panel shows the viability of cells at 144 h after transfection **e** Phosphorylation profile of major RTKs signaling in IGF2R-knockdown cells at 72 h after siRNA transfection. A complete target map is shown in the bottom panel. **f** Time-dependent changes in p38 MAPK phosphorylation status after IGF2R knockdown. **h** Rescue effect of p38 MAPK inhibition on IGF2R knockdown-induced cell death. siRNA-transfected cells were further exposed to 1 μM SB203580 (p38 MAPK inhibitor) for 96 h, and their viabilities were measured. All the error bars represent the standard deviation of three independent experiments. *p *<* 0.05, **p *<* 0.01, ****p *<* 0.0001; Dunnett’s multiple comparisons test. Scale bars represent 20 μm.

Since IGF2R is widely accepted as a scavenger of IGF-2, which activates IGF1R signaling, we verified whether IGF2R knockdown-induced apoptosis could be attributed to alterations in IGF1R signaling. Immunocytochemical analyses showed that IGF-2 bound to IGF2R on cervical cancer cells and other cancer cells, as previously reported (Fig. 3a). However, the translocation of IGF-2 into lysosomes was not inhibited by IGF2R knockdown (Fig. 3b). Neither IGF2R knockdown nor IGF-2 addition activated IGF1R signaling pathways (Fig. 3c). Furthermore, IGF1R knockdown did not rescue the cell death induced by IGF2R knockdown (Fig. 3d). This indicates that IGF-2–IGF1R signaling is not particularly significant in cervical cancer cells. Besides IGF1R, the major receptor tyrosine kinase signaling pathways were not altered by IGF2R knockdown (Fig. 3e). Further signaling analysis revealed that IGF2R knockdown activated a stress-activated serine/threonine protein kinase, p38 mitogen-activated protein kinase, in a time-dependent manner (Fig. 3f), whereas inhibition of the kinase did not rescue IGF2R knockdown-induced cell death (Fig. 3g), suggesting an indirect signal activation associated with cell death. Therefore, we considered that novel mechanisms of oncogenic IGF2R may exist in cervical cancer.

### Downregulation of Golgi apparatus-centered vesicular transport by IGF2R knockdown

**Fig. 4:**
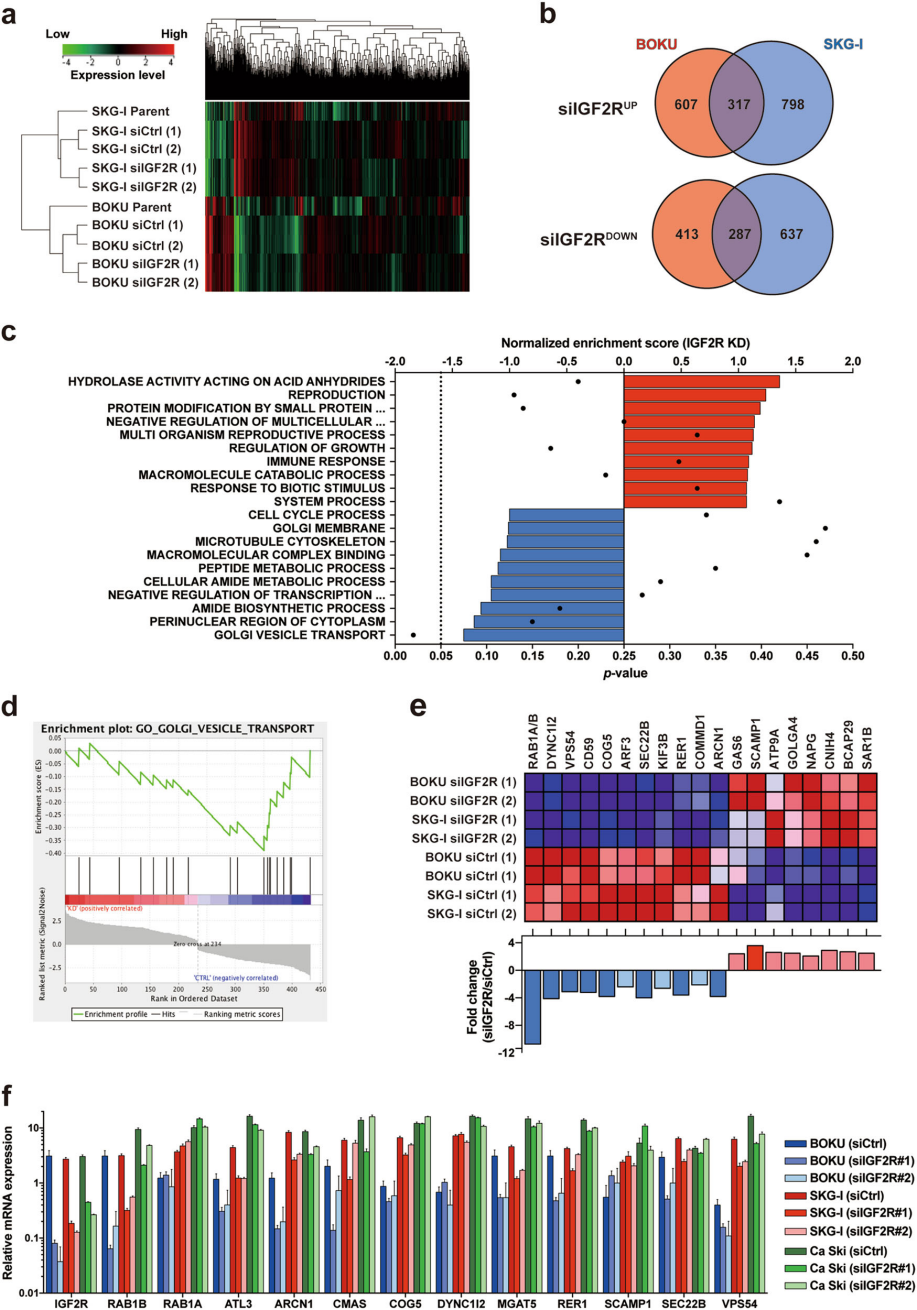
Transcriptomic analysis in IGF2R-knockdown cervical cancer cells. **a** An unsupervised hierarchical clustering based on gene expression. The same total RNA from siRNA-transfected cells were analyzed in duplicate (i.e., (1) and (2)). **b** IGF2R-regulated genes in cervical cancer cells. Venn diagrams of probes for which expression was increased (top panel) or decreased (bottom panel) by IGF2R knockdown. The number of probes in each group is shown in the circles. A list of the probes common among cell lines is shown in Supplementary Table 1. **c** Gene set enrichment analysis (GSEA) of a probe set that commonly changed their expression after IGF2R knockdown. Top ranked gene ontology terms are shown in the graph. Bars and plots depict the normalized enrichment score and *p*-value of each gene ontology term, respectively. A vertical dashed line represents *p* *=* 0.05. **d** Enrichment plot of a gene ontology term, “GOLGI VESICLE TRANSPORT,” which is the only gene set with significant *p*-values in the GSEA. **e** Expression profiles of the 19 genes included in the gene set in (**d**). The mRNA expression levels of the genes in siRNA-transfected samples are shown as a heat map (top panel). Red and blue columns represent increased and decreased expression of a gene, respectively. The fold change values between the groups in (**c**) are shown in the bottom panel. **f** Quantification of the mRNA expression level of genes included in the gene set in (**d**). Two kinds of siRNAs targeting IGF2R (#1 and #2) were used. Error bars depict the standard error.

Transcriptome analyses in IGF2R-knockdown cervical cancer cells (Fig. 4a) identified 317 and 287 genes to be commonly upregulated or downregulated, respectively, by IGF2R knockdown (Fig. 4b, Supplementary Table S1, and GEO accession number: GSE137998). Although gene set enrichment analysis identified both positively and negatively enriched gene set candidates (Fig. 4c), a gene set relevant to vesicle transport in the Golgi apparatus was the only significant result among them. Overall, IGF2R knockdown decreased the expression of 11 genes in the gene set (Fig. 4d–f), suggesting that the receptor participates in the regulation of vesicle transport in the Golgi apparatus.

### IGF2R maintains autophagy and mitophagy by regulating lysosomal homeostasis

**Fig. 5:**
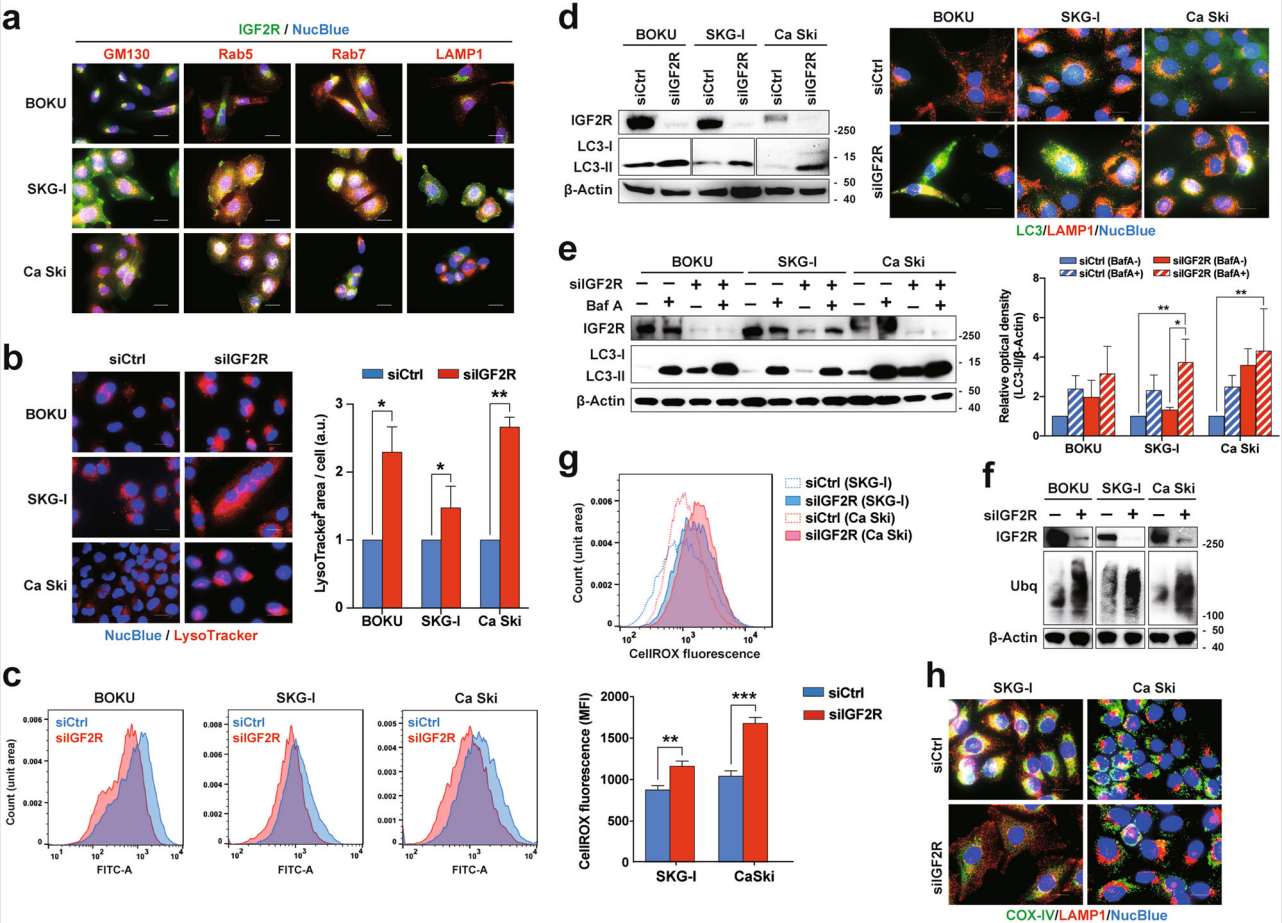
IGF2R maintains autophagy and mitophagy through lysosome homeostasis. **a** Distribution of IGF2R in subcellular compartments. GM130, Rab5, Rab7, and LAMP1 are markers for the Golgi apparatus, early endosome, late endosome, and lysosome, respectively. **b** Accumulation of acidic organelles in IGF2R-knockdown cells at 96 h after siRNA transfection. The LysoTracker^+^ area in the cells was measured and normalized by the number of nuclei. **c** Activities of lysosomal enzymes in IGF2R-knockdown cells. The activities were assessed using a self-quenching fluorophore-labeled peptide probe, which yields green fluorescence in response to lysosomal enzyme cleavage. Flow cytometric analysis of the probe-treated cells at 96 h after siRNA transfection. **d** Induction of autophagosome formation by loss of IGF2R. Western blot analysis of the LC3-I and LC3-II status in cells at 96 h after siRNA transfection (left panel). Immunocytochemical analysis of autophagosomes and autolysosomes in the cells under the same conditions (right panel). Anti-LC3 and anti-LAMP1 antibodies were used to detect autophagosomes (green) and lysosomes (red), respectively. **e** Effect of autophagic flux on IGF2R knockdown. Western blot analysis shows the conversion of LC3-I to LC3-II in cells with siRNA transfection (96 h), followed by treatment with an inhibitor of lysosome acidification, Bafilomycin A1 for 24 h (left panel). Densitometry analysis from three independent experiments (right panel). **f** Western blot analysis of ubiquitinylated proteins in IGF2R-knockdown cells at 72 h after siRNA transfection. **g** Reactive oxygen species (ROS) production in cells with IGF2R knockdown. The amount of intracellular ROS was analyzed by CellROX reagent, which yields fluorescence by oxidation. Representative histogram of flow cytometry (top panel) and its mean fluorescent intensity (bottom panel). **h** Activities of mitophagy in cells with a loss of IGF2R. Representative images of cells under knockdown of IGF2R at 96 h after transfection. Mitochondria and lysosomes in the cells were stained by anti-COX IV (green) and anti-LAMP1 (red) antibodies, respectively. All the error bars represent the standard deviation of three independent experiments. *p *<* 0.05, **p *<* 0.01, ***p *<* 0.001; *t-*test with Welch’s correction and Tukey multiple comparisons test. Scale bars represent 20 μm.

IGF2R binds to M6P-labeled proteins and transports them from the trans-Golgi network (TGN) to the lysosome via vesicular transport systems^16^. Moreover, over 90% of the receptor localizes around intracellular organelles, such as Golgi apparatuses and late endosomes^17,18^. Consistent with these findings, most of the IGF2R was localized intracellularly and co-localized with Golgi apparatuses and early/late endosomes but not with lysosomes (Supplementary Fig. S3a and Fig. 5a). Next, we validated lysosome function in IGF2R-knockdown cells, since lysosomes are considered to be the organelles most affected by the insufficient delivery of M6P-labeled lysosomal enzymes. Time-dependent accumulation of both acidic and LAMP1-positive organelles was observed in IGF2R-knockdown cells (Fig. 5b and Supplementary Fig. S3b, c), suggesting that the receptor maintains lysosome homeostasis. Increased lysosomal membrane permeability (LMP) induces apoptosis via the release of lysosomal enzymes to the cytoplasm;^19^ however, IGF2R knockdown did not induce LMP (Supplementary Fig. S3d). In contrast, the activities of lysosomal enzymes were decreased by IGF2R depletion (Fig. 5c and Supplementary Fig. S3e). Lysosomes are inseparably related to various types of autophagy such autophagy and mitophagy^20^. Here, IGF2R knockdown promoted autophagosome formation (Fig. 5d). Considering that bafilomycin A-induced LC3-II conversion was not enhanced by IGF2R knockdown, as evidenced by densitometry analysis of three independent experiments, this knockdown had an autophagic inhibitory effect (Fig. 5e). Accordingly, polyubiquitinylated proteins were observed to be accumulated in IGF2R-depleted cells (Fig. 5f). Besides, IGF2R knockdown substantially reduced the mitochondrial membrane potential, indicating an accumulation of damaged mitochondria in the cells (Supplementary Fig. 3f). As a result, ROS production was augmented in IGF2R-knockdown cells (Fig. 5g). Immunocytochemical analysis showed a loss of mitophagosomes in IGF2R-knockdown cells (Fig. 5h), suggesting that IGF2R is essential for autophagy and mitophagy.

### IGF2R has a dominant role in the effective transportation of cathepsins to lysosomes

**Fig. 6:**
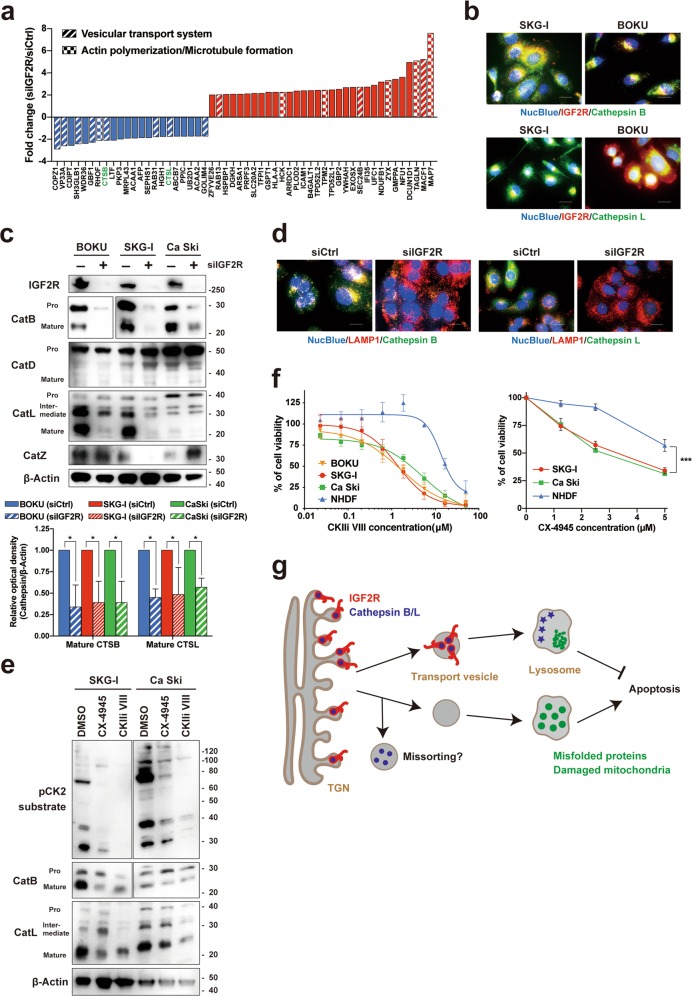
CK2 regulates cathepsin transportation via IGF2R phosphorylation. **a** Proteomic analysis of IGF2R-knockdown cells (SKG-I and CaSki) at 96 h after siRNA transfection. Each fold change value was calculated by digitizing the peak intensity obtained from mass spectrometry. The currently known M6P-labeled protein is colored green. **b** Co-localization of cathepsins and IGF2R in cervical cancer cells. Parental cells were co-stained with anti-cathepsin B/L (green) and anti-IGF2R (red) antibodies. **c** Effect of IGF2R knockdown (72 h) on protein expression and processing of cathepsins. Western blotting (upper panel) and densitometry analysis of matured cathepsin B and L expression from three independent experiments (lower panel). **d** Changes in the intracellular distributions of cathepsins after IGF2R knockdown (72 h). **e** Effect of CK2 inhibitors on the protein expression and processing of cathepsins. Protein expression in CK2 inhibitor-treated cells (72 h) were analyzed by western blotting. **f** Dose-response effects of CK2 inhibitors on the cell viability of cervical cancer cells and normal human dermal fibroblasts (NHDFs). Viable cells were detected at 96 hours after the treatment. **g** Schematic diagram of the biological significance of IGF2R in cervical cancer cells. All the error bars represent the standard deviation of three independent experiments. *p *<* 0.05, ***p *<* 0.001; *t*-test with Welch’s correction and Dunnett’s multiple comparisons test. Scale bars represent 20 μm.

Mass spectrometry-based comprehensive proteomic analysis showed that IGF2R knockdown downregulated many proteins relevant to vesicular transport systems, consistent with the transcriptome analysis (Fig. 6a). Notably, two M6P-labeled lysosomal enzymes (cathepsin B and L) were downregulated by IGF2R knockdown. Under normal conditions, both cathepsin B and L are partly co-localized with IGF2R in cervical cancer cells (Fig. 6b). Analysis of external RNAseq data showed that four members of the cathepsin family (*CTSD*, *CTSB*, *CTSZ*, and *CTSL*) are relatively more highly expressed (Supplementary Fig. S4a). Moreover, proteome data showed that all the cathepsins were downregulated in IGF2R-knockdown cells (Supplementary Fig. S4b). The RNAseq data also revealed significantly poorer survival of patients with cervical cancer when related to high *CTSB* and *CTSZ* expression (Supplementary Fig. S4c). There was no correlation between the mRNA expression level of IGF2R and that of cathepsins (Supplementary Fig. S4d). The mRNA expression of cathepsins was not influenced much by the loss of IGF2R (Supplementary Fig. S4e). In contrast, the protein expression levels of cathepsin B and cathepsin L were significantly reduced by IGF2R knockdown (Fig. 6c, d). It is noteworthy that the loss of IGF2R downregulated the protein expression of mature cathepsins but showed a lower effect on their mRNA expression, suggesting the failure of post-transcriptional intracellular transportation of these proteins from the TGN to the lysosome. Considering that IGF2R knockdown suppressed lysosomal activity, the incomplete transportation of these cathepsins might be a leading cause of IGF2R depletion-induced apoptosis. The abnormal release of intracellular proteins is another probable cause of apoptosis, since most lysosomal hydrolases are secreted to extracellular regions in M6P receptor-deficient cells^16^. However, the secreted factors from the IGF2R-knockdown cells did not inhibit cell growth (Supplementary Fig. S4f). Cation-dependent mannose-6-phosphate receptor (CD-M6PR, hereafter M6PR) has also been reported as a major M6P receptor^21^. We further investigated the relationship between IGF2R and M6PR in cervical cancer cells. DNA microarray analysis showed that M6PR was not aberrantly expressed in cervical cancer tissues (Supplementary Fig. S4g). Furthermore, the mRNA expression levels of M6PR did not impact the prognosis of cervical cancer patients (Supplementary Fig. S4h). In contrast to IGF2R, M6PR knockdown did not influence intracellular cathepsins, protein ubiquitinylation, or cervical cancer cell survival (Supplementary Fig. S4i, j).

### Blockade of CK2 suppressed IGF2R-mediated transportation and induced cancer cell death

CK2 phosphorylates the cytoplasmic tail of IGF2R and M6PR, which is critical to form complexes with clathrin adaptor proteins, and this is followed by trafficking^22–24^. To clarify the inhibitory effect of CK2 on IGF2R, we analyzed the modulation of the subcellular localization of IGF2R by the CK2 inhibitors CX-4945 (silmitasertib) and CK2 inhibitor VIII. As expected, treatment with these compounds increased the localization of IGF2R in the Golgi apparatus and decreased it in late endosomes (Supplementary Fig. 4l). Similar to IGF2R knockdown, the protein expression levels of matured cathepsin B and L were reduced in CK2 inhibitor-treated cells, with effective inhibition of CK2 activity (Fig. 6e). Furthermore, a dose-dependent accumulation of acidic organelles was observed (Supplementary Figs. S4k), which had the same phenotypes as shown in the IGF2R-knockdown cells. Notably, CK2 inhibitors selectively inhibited the survival of cervical cancer cells compared to normal human dermal fibroblasts (NHDFs) (Fig. 6f). These results suggest that pharmacological inhibition of CK2 can regulate the oncogenic functions of IGF2R in cervical cancer cells; however, this study did not show direct interaction of IGF2R and CK2 (e.g. phosphorylation of IGF2R by CK2). Further studies are needed in order to develop therapeutic strategies based on the CK2-IGF2R axis.

## Discussion

The discovery of new biomarkers and therapeutic targets for achieving stratified and precision medicines is becoming increasingly important. Unfortunately, in cervical cancer, the use of conventional tumor markers such as SCC, CEA, and CA-125 remains the dominant practice^25^. A recent integrated omics approach revealed that patients with cervical cancer can be classified into clinically relevant subgroups by their genomic and proteomic characteristics (e.g., activation of a PI3K pathway)^26^. Current clinical trials have demonstrated that the administration of PI3K or mTOR inhibitors to patients with *PI3KCA*-mutated cervical cancer is effective^27^. These previous reports strongly support a promising future for individual medicine in cervical cancer; however, the number of usable biomarkers is currently limited.

Here, we identified IGF2R as a novel biomarker for poor prognosis in patients with cervical cancer. While IGF2R is ubiquitously expressed in human tissues^28^, the LOH of the chromosome locus and a reduction in protein expression were frequently observed in various types of cancer^8–11,29^. This evidence underpins the current shared understanding of *IGF2R* as a tumor suppressor gene. With regard to cervical cancer, however, IGF2R expression was increased in cancer tissues (Fig. 1c, e), and patients with high IGF2R expression had worse prognoses (Fig. 1f). IGF2R expression has been associated with recurrence-related prognoses in primary glioblastoma tumors^30^. Thus, in some cancers, *IGF2R* may behave not as a tumor suppressor gene but as an oncogene. This was also indicative from survival analysis: high *IGF2R* expression was correlated with poor prognosis in several types of cancers, while *IGF2R* might function as a tumor suppressor in cancers where high IGF2R expression is correlated with good prognosis (Supplementary Fig. S1e). Surprisingly, our RNAseq data analysis showed that IGF2R is also a poor prognostic factor for early-stage cervical cancer (Fig. 1f), suggesting its role in tumor recurrence. In addition to recent surgical developments, the clinical outcomes of adjuvant treatments have recently improved for early-stage cervical cancer. Therefore, pre-emptive medical care may yield clinical benefits for early-stage cervical cancer patients with high IGF2R expression.

Our gene knockdown studies revealed that IGF2R is essential for cell survival and that cell viability decreases with time (Fig. 2b, c). The cell viabilities of SKG-IIIa and HCS-2 were not significantly affected by IGF2R knockdown, which might be due to insufficient knockdown of IGF2R in HCS-2. Meanwhile, IGF2R knockdown was not significant but tended to decrease the viability of SKG-IIIa cells. IGF1R expression was also affected by IGF2R knockdown, especially in BOKU cells (Fig. 2b). However, validation analysis revealed that a single IGF2R knockdown was sufficient to reduce cell viability and colony formation and induce sensitivity to cisplatin without a change in IGF1R expression (Supplementary Fig. S2f–h). Moreover, loss of IGF2R induced caspase-mediated apoptosis in cervical cancer cells (Fig. 2d, e). Interestingly, these observations contradict those of a study on non-small cell lung cancer^31^. Meanwhile, another study reported a loss of IGF2R-induced apoptosis in hemangioma cells^32^. Thus, the two-faced traits of IGF2R might depend on cell type. Since the present RNAseq data analysis showed high IGF2R expression to be associated with poor prognosis in some gynecological cancers (Supplementary Fig. S1e), further studies will be needed to elucidate the oncogenic function of IGF2R in these cancers.

The main biological function of IGF2R is the suppression of IGF1R signaling via the deprivation of extracellular IGF-2 ligands. Some studies explained the tumor suppressive functions of IGF2R by its negative regulation of the oncogenic IGF-2–IGF1R signal axis^33^. However, our study shows that, in cervical cancer cells, loss of IGF2R did not alter the major receptor tyrosine kinase pathways (Fig. 3c, e). Phosphorylation of Akt and S6 ribosomal protein seemed to be reduced in some cell lines; however, these changes were not universal, and the pattern of Akt phosphorylation was not uniform in different cell lines by western blot analysis (Fig. 3c). Considering that IGF1R knockdown did not increase apoptosis (Fig. 2d), IGF-2–IGF1R signaling is not necessary for cervical cancer cells to survive. Alternatively, IGF2R knockdown induced the accumulation of abnormal lysosomes with lower proteolytic activity (Supplementary Fig. S3c and Fig. 5c). Accordingly, IGF2R plays a critical role in lysosome homeostasis by maintaining Golgi-to-lysosome transportation. Lysosomes are inextricably tied to autophagy, an important process for the degradation and recycling of intracellular components. From autophagic flux analysis, bafilomycin A-induced LC3-II conversion was not enhanced by IGF2R knockdown, suggesting that this knockdown had an autophagic inhibitory effect. Meanwhile, LC3-II expression in the IGF2R knockdown background was enhanced by bafilomycin A, indicating that the autophagic inhibitory effect of IGF2R knockdown was not as strong as that of bafilomycin A (Fig. 5e). This notion was also supported by the accumulation of polyubiquitinylated proteins and ROS, which should have been regulated by autophagy and mitophagy (Fig. 5f)^34,35^. These sequential failures may lead to apoptosis-mediated cell death in cervical cancer cells. These points highlight that the effect on cell viability takes time to manifest; hence, we decided on detecting cell viability 144 h after siRNA transfection.

Among lysosomal enzymes, cathepsins are key acid hydrolases involved in the endolysosomal organelle system. They participate in nearly all processes associated with lysosomes, such as autophagy. Cathepsins are synthesized as inactive pro-cathepsins and are then post-translationally modified by glycosylation. During this process, M6P is necessary for translocation to the lysosome via an interaction with IGF2R^36^. Our IGF2R knockdown experiments clearly induced a loss of cathepsin expression, especially cathepsin B and L (Fig. 6c). Cathepsins have been found to be extracellularly secreted in M6P receptor-deficient cells^16,37^. Therefore, IGF2R depletion might cut off the supply of immature cathepsins to lysosomes for maturation, releasing the accumulated immature cathepsins to the extracellular space via machinery different from that of M6PR-mediated transportation (e.g., a secretory pathway). As a result of their missorting, both mature and immature cathepsins decrease. We tried to show the direct interaction of IGF2R and cathepsins by IGF2R overexpression using cDNA and lentiviral vectors or pull-down assays; however, we were unsuccessful, probably due to the large size of IGF2R (275 kDa) and lack of appropriate antibodies. Neither decreased cathepsin expression nor decreased cell survival was observed in M6PR-knockdown cells, although M6PR is also known to transport M6P-labeled cargo (Supplementary Fig. S4i, j)^36^. Therefore, IGF2R blockade could be critical to cervical cancer cells, and the blockade of this Golgi-to-lysosome transport system could be a potential therapeutic target.

IGF2R has been reported to have four phosphorylation sites in its cytosolic tail, which are necessary for IGF2R-mediated trafficking^36^. After phosphorylation, clathrin adaptor proteins are recruited on the phosphorylation site, finally budding from TGN using clathrin-mediated machinery^21–23^. Importantly, this phosphorylation is catalyzed by CK2, a serine/threonine kinase^24,38^. CK2 is also known to regulate the cell cycle and several signaling pathways, such as the Wnt pathway^39^. In cervical cancer, CK2 activity is higher in HPV-immortalized cell lines compared to normal keratinocytes, and it is regulated by the HPV E7 viral oncoprotein^40^. In fact, CK2 inhibitors kill cervical cancer cells; CIGB-300, a CK2 inhibitor, has been found to suppress the proliferation of cervical cancer cells and show synergistic effects with paclitaxel and doxorubicin^41^. Our study shows that both CK2 inhibitors (CX-4945 and CKII inhibitor VIII) suppressed IGF2R translocation from the TGN to the endosomes (Supplementary Fig. S4l). Furthermore, these inhibitors also induced the accumulation of the acidic organelle and the downregulation of mature cathepsin B and L, as observed in IGF2R-knockdown cells (Supplementary Fig. S4k and Fig. 6e). The expression of “pro” and “intermediate” cathepsins differed slightly by siIGF2R and CK2 inhibition; “pro” and “intermediate” cathepsins were downregulated in IGF2R-knockdown cells but were not affected much in CK2-inhibited cells. However, these expression patterns also differed by the types of CK2 inhibitors. CK2 inhibitors may affect not only IGF2R but also another M6PR, and these differences might be the result of blockade of CK2 or an inhibitor-specific effect on the trafficking of “pro” and “intermediate” cathepsins. Considering the IGF2R functions demonstrated in this study, the selective killing ability of the inhibitors might be attributed to the suppression of IGF2R functioning (Fig. 6f, Supplementary Fig. S4k, l). We tried to show the direct relationship between CK2 and IGF2R by detecting the direct phosphorylation of IGF2R by CK2 inhibitors and CK2 knockdown; however, this was not achieved because it is difficult to detect its phosphorylation and knockdown the entire CK2. Alternatively, we investigated the similarity of IGF2R knockdown and CK2 inhibition to show the indirect CK2-IGF2R axis. CX-4945 and CIGB-300 have been in clinical trials in several past and ongoing cancer studies, including cervical cancer^42,43^. Our results showed that CK2 inhibitors are promising drugs targeting not only the cell cycle and several signaling pathways but also IGF2R-mediated M6P-cargo transport, warranting further challenging but beneficial investigations.

In conclusion, we have shown that high IGF2R expression is correlated with poor prognosis in cervical cancer. IGF2R has a role opposite to its currently recognized tumor suppressive role, and its function as a transporter of M6P cargo to maintain lysosome homeostasis is important in cervical cancer cells (Fig. 6g). Therefore, targeting IGF2R itself, its M6P-tagged cargo, or Golgi-to-lysosome transport by CK2 inhibitors may be potential therapeutic strategies. Furthermore, the expression of IGF2R can be used as a prognostic biomarker for cervical cancer patients.

## Materials and methods

### Clinical samples

Cervical cancer tissue samples were obtained from patients undergoing surgery at Keio University Hospital (Tokyo, Japan). All six patients had given written informed consent for the study protocol, which was approved by the ethics committee of Keio University (No. 2007-0081, 2013-0336). Experiments on the tissue samples were conducted in accordance with the approved guidelines.

### External data analysis

Both normalized RNA sequence data (RNA Seq V2 RSEM) and their corresponding clinical information of cervical squamous cell carcinomas and endocervical adenocarcinomas (306 cases) were downloaded from The cBioPortal for Cancer Genomics website (*cBioPortal*, http://www.cbioportal.org/). Integration analyses (OncoPrint) of gene mutations, copy-number alterations, and mRNA expression, as well as co-expression analyses of genes-of-interest (GOI), were executed by the website using same dataset. In each GOI, samples were classified by FPKM value, and the overall survival of each group was analyzed using the Kaplan–Meier method and log-rank tests. In some GOIs, a limited number of samples (291 cases) from the above dataset, all of which are available from The Human Protein Atlas website (*Protein Atlas*, https://www.proteinatlas.org/), were used for survival analyses. All FPKM values ranging from the 20th to the 80th percentile was used to classify and examine the significant differences in overall survival by the log-rank test. Among them, the group that showed the lowest log-rank *p*-value was used this study. Overall survival of the GOI in another 16 cancers, which can be downloaded from the *Protein Atlas* website, were also analyzed. Through the Gene Expression Omnibus website (https://www.ncbi.nlm.nih.gov/geo/), a DNA microarray dataset (GDS3233) including normal cervix (24 cases) and cervical cancer (28 cases) samples was downloaded, and the normalized mRNA expression values of the GOI were analyzed. Normalized mRNA expression data of IGF2R were also downloaded from ArrayExpress (GEOD-27678, https://www.ebi.ac.uk/arrayexpress/), which included cervical cancer biopsy samples from patients before and after receiving radiotherapy (20 cases) or chemoradiotherapy (19 cases).

### Cell lines and cell cultures

Human cervical cancer cell lines (BOKU, SKG-I, SKG-II, SKG-IIIa, HCS-2, and Ca Ski) and primary NHDFs were purchased from the Japanese Collection of Research Bioresources Cell Bank and the Cell Systems Corporation, respectively. BOKU, SKG-I, SKG-II, and SKG-IIIa cells were cultured in Ham’s F12 (Wako, Osaka, Japan); HCS-2 was cultured in EMEM (Wako); Ca Ski was cultured in RPMI-1640 (Wako); and NHDFs were cultured in DMEM (Wako) with 10% fetal bovine serum (FBS) (Gibco, Waltham, MA, USA) and 100 U/mL of penicillin-streptomycin (Gibco). All cell lines were maintained at 37 °C with 5% CO_2_. To avoid cross-contamination and incorrect authentication, all cell lines were used for experiments within 4 years of purchase.

### Drugs and antibodies

The chemical compounds used in this study included cisplatin (Wako), bafilomycin A1, CX-4945 (AdipoGen, CA, Switzerland), CKII inhibitor VIII (Merck Millipore, Burlington, MA, USA), and SB2035801 (Cayman Chemical, Ann Arbor, MI, USA). Anti-cathepsin D (C-5), anti-cathepsin Z (F-6), and anti-ubiquitin (F-11) antibodies were obtained from Santa Cruz Biotechnology (Dallas, TX, USA). Anti-IGF2R (D8Z3J), anti-IGF1R (D23H3), anti-phospho-IGF1R, anti-Rab5 (C8B1), anti-LAMP1 (D4O1S), anti-LC3A/B (D3U4C), Alexa Fluor 488-conjugated anti-COX ΙV(3E11), anti-cathepsin B (D1C7Y), anti-p44/42 MAPK (137F5), anti-phospho-p44/42 MAPK (Thr202/Tyr204, D13.14.4E), anti-Akt (C67E7), anti-phospho-Akt (S473, D9E), anti-p38 MAPK (D13E1), anti-phospho-p38 MAPK (Thr180/Tyr182, D3F9), anti-CK2 substrate [(pS/pT)DXE] MultiMab mix, and anti-β-actin antibodies were purchased from Cell Signaling Technology (Danvers, MA, USA). Anti-IGF-2, anti-GM130, anti-Rab7 (EPR7589), and anti-M6PR (EPR7691) antibodies were purchased from Abcam (Cambridge, UK). Anti-cathepsin L antibodies were purchased from Proteintech (Rosemont, IL, USA). An anti-IGF2R antibody (used for the immunohistochemical analysis) was purchased from Sigma-Aldrich (St, Louis, MO, USA). An Alexa Fluor 647-conjugated anti-LAMP1 antibody was purchased from Biolegend (San Diego, CA, USA). Alexa Fluor 488-conjugated anti-rabbit IgG and Alexa Fluor 594-conjugated anti-rabbit IgG were purchased from Invitrogen (Waltham, MA, USA). HRP-labeled anti-rabbit immunoglobulin antibodies were obtained from Dako (Santa Clara, CA, USA).

### Transient gene knockdown

All the siRNAs used this study were purchased from Thermo Fisher Scientific (MA, USA): IGF2R (s7219, HSS105256, s7217, s7218, HSS105257, and HSS105258), IGF1R (s7211), and M6PR (s8375). Transfections were performed using a transfection reagent, DhamaFECT1 (GE Healthcare, Chicago, IL, USA), in accordance with the manufacturer’s instructions. Briefly, siRNA and DharmaFECT1 were suspended in OptiMEM (Gibco), followed by the addition of the cultured cells to a final concentration of 10 nM. After culturing for 24 h, the medium was exchanged with normal medium, and the cells were used in downstream assays. In combinatorial siRNA transfection experiments, 10 nM of siCtrl and 10 nM of target siRNA were mixed to adjust the total amount of siRNA for combinatorial siRNA mixture (10 nM of the first siRNA with 10 nM of the second siRNA).

### Viability assays

End point detections of cell viability were mainly achieved using the Cell Counting Kit-8 (Dojinkagaku, Kumamoto, Japan) or CellTiter-Glo 2.0 Assay (Promega, WI, USA). Absorbance at 450 nm (A_450_) or luminescence was measured by a Synergy H1 microplate reader (BioTek, Winooski, VT, USA). According to the instructions, a RealTime-Glo MT Cell Viability Assay kit (Promega) was used to monitor cell viability over time. After the addition of RealTime-Glo reagent, cells were cultured at 37 °C with 5% CO_2_ in a Synergy H1 microplate reader, and the chemiluminescence was detected every 30 min.

### 3D culture

In this study, cell growth and/or death was detected in two different 3D culture assays: using Matrigel (Corning, Corning, NY, USA) and low attachment plates, respectively. The Matrigel-based 3D culture assay was performed according to the protocol of 3D on-top assays, as reported by Lee et al.^44^. siRNA-treated cells were resuspended with 10% Matrigel-containing ice-cold growth medium at a concentration of 2 × 10^5^ cells/mL. Samples (250 μL) of the mixtures were then added to the Matrigel pre-coated 24-well plate carefully coated with 250 μL of Matrigel and cultured at 37 °C with 5% CO_2_ for 96 h. Colonies in the Matrigel were photographed and counted by ImageJ software. The second 3D culture assay was performed using a low adherent plate. Briefly, 1 × 10^4^ of siRNA-transfected cells were reseeded into the EZ Bind Shut II, a round-bottomed 96-well plate (IWAKI, Shizuoka, Japan) and cultured at 37 °C with 5% CO_2_ for 12 days. Viable and dead cells in the spheroids were simultaneously visualized by the LIVE/DEAD Viability/Cytotoxicity Kit (Thermo Fisher Scientific), according to the supplier’s protocol. Furthermore, the cell viability of the spheroids was detected using the CellTiter-Glo 3D Cell Viability Assay (Promega) according to the manufacturer’s protocol.

### Apoptosis detection

Apoptosis was detected by the FITC Annexin V Apoptosis Detection Kit (BD Bioscience, CA, USA). Briefly, cells were resuspended in AnnexinV Binding Buffer and co-stained with FITC-AnnexinV and 7-AAD for 15 min at room temperature. Cells were then analyzed by BD FACSVerse (BD Biosciences), and apoptotic cells were calculated as the sum of the early apoptotic (AnnexinV^High^/7-AAD^Low^) and late apoptotic (AnnexinV^High^/7-AAD^High^) cell populations. Similarly, the activities of caspase 3 and 7 in the cultured cells were also detected using Caspase-Glo 3/7 Assay Systems (Promega) according to the manufacturer’s protocol. The chemiluminescent intensity of each sample was measured using a Synergy H1 microplate reader after the addition of Caspase-Glo 3/7 reagent to cells cultured in opaque 96-well plates.

### Wound healing assay

Cells were seeded in 6-well plates at a high density (4 × 10^5^ cells/well), followed by siRNA transfection and culturing overnight. Confluent monolayer cells were linearly scratched using a CELL Scratcher (Asahi Technoglass, Shizuoka, Japan). After cells were washed with PBS, they were cultured in normal growth medium for 48 h. The cell scratches were photographed, and the scratched area was measured by ImageJ software.

### Invasion assay

BD BioCoat Matrigel Invasion Chambers (BD Bioscience) were used to validate cellular invasion abilities. Hydrated invasion chambers were set on 24-well companion plates (BD Bioscience) filled with 10% FBS-containing growth medium. The siRNA-transfected cells were then resuspended with FBS-free growth medium and 400 μL of the suspension added into the chambers at a density of 1 × 10^5^ cells/well. Following culturing for 48 h, the membranes of the chambers were fixed using Diff-Quick fixing agent and then stained with Diff-Quick solutions 1 and 2 (SYSMEX, Hyogo, Japan) for 2 min each. After cells were washed in PBS, the stained cells on the membranes were photographed by microscopy and counted using ImageJ software.

### Cell cycle analysis

Cultured cells were resuspended with ice-cold 80% ethanol, followed by incubation at −30 °C overnight. After cells were washed in PBS, they were incubated in PI/RNase staining buffer (BD Bioscience) on ice for 1 h, and their cell cycle profiles were analyzed using BD FACSVerse.

### Phospho-kinase array

The phosphorylation of biologically important receptor tyrosine kinases (28 kinases) and their downstream molecules (11 nodes) in the cultured cells was validated using the sandwich immunoassay-based PathScan RTK Signaling Antibody Array Kit (Cell Signaling Technology) according to the manufacturer’s instructions. Finally, the luminescent signal of each spot was detected using the ImageQuant LAS 4000 mini system (GE Healthcare).

### Western blot analysis

Proteins were extracted in 1X Laemmli Sample Buffer (Bio-Rad Laboratories, Hercules, CA, USA) containing 350 mM DTT and 1% protease inhibitor cocktail (Sigma-Aldrich). The protein concentration of each sample was measured by Protein Quantification Assay (Macherey-Nagel, Nordrhein-Westfalen, Germany). Approximately 10–30 μg protein and MagicMark XP Western Protein Standard (Thermo Fisher Scientific) were electrophoresed by NovexWedgeWell 4–20% Tris-Glycine Gel (Thermo Fisher Scientific) under 225 V for 30 min. The proteins were then transferred to Immobilon-P PVDF membranes (Merck Millipore) under 100 mA for 40 min using the Trans-Blot Turbo Blotting System (Bio-Rad Laboratories). Membranes were incubated with PBS (Blocking Buffer) containing 5% Membrane Blocking Agent (GE Healthcare) for 2 h and then reacted with the optimal concentrations of primary antibodies overnight at 4 °C. After membranes were washed in PBS with 0.05% Tween 20 (PBST), they were reacted with the corresponding HRP-labeled secondary antibodies for 1 h. To reduce non-specific binding of antibodies, Can Get Signal Immunoreaction Enhancer Solution (Toyobo, Osaka, Japan) was used to dissolve primary/secondary antibodies as a substitute for blocking buffer in some cases. After proteins were washed in PBST, the proteins on the membranes were visualized by the ImageQuant LAS 4000 mini system (GE Healthcare) and FUSION system FX6.EDGE (VILBER LOURMAT, Collegien, France) using Pierce Western Blotting Substrate plus (Thermo Fisher Scientific). All the experiments were performed at least twice for confirmation, and some experiments were performed three times with quantification, normalization, and statistical analysis.

### Quantitative RT-PCR

Total RNA was isolated from cultured cells using ISOGEN (Nippon Gene, Tokyo, Japan), followed by isopropanol precipitation-based purification. cDNA was then synthesized using the SuperScript IV VILO Master Mix (Thermo Fisher Scientific), according to the manufacturer’s instructions. Real-time PCR assays were performed by the IQ SYBR Green Supermix (Bio-Rad Laboratories) on a CFX96 Real-Time PCR Detection System (Bio-Rad Laboratories). Details of the primers used in this study are described in Supplementary Table S2.

### Microarray analysis

Total RNA was analyzed by the GeneChip Human Genome U133 Plus 2.0 arrays (Affymetrix, Santa Clara, CA, USA) that included 54,675 probe sets for the analysis of the mRNA expression levels of approximately 47,000 transcripts and variants from 38,500 well-characterized human genes. The procedures for target hybridization, washing, and staining with signal amplification were conducted according to the supplier’s protocols. The arrays were scanned with a GeneChip Scanner 3000 (Affymetrix), and the intensity of each feature of the array was calculated in GeneChip Analysis Suite version 4.0 software (Affymetrix). The mean expression value in each experiment was normalized to 1,000 to reliably compare multiple arrays. Entities in which 90% of the samples had values under 500 were excluded from the analyses. Using the expression data of the filtered entities, hierarchical clustering was performed using GeneSpring (Agilent Technologies, Santa Clara, CA, USA). To identify genes whose mRNA expression levels differed between the control and treated groups, we selected entities with over a 2-fold change and further filtered the results using unpaired *t*-tests (*p* *<* 0.05). Using the entities selected as explained above, GSEA was performed by GSEA v2.06 (downloaded from the Broad Institute website, http://software.broadinstitute.org/gsea/index.jsp). In the analysis, the GO gene sets (C5 collection) containing 5917 genes were used as the gene set database.

### Mass spectrometric analysis

Comprehensive proteomics data were acquired by a two-dimensional image-converted analysis of liquid chromatography and mass spectrometry (2DICAL) shotgun proteomics analysis, as described previously^45,46^.

### Immunocytochemistry

After treatment with various reagents (e.g., siRNA or drugs) in 24-well plates, cells were reseeded in 8-well non-coating slide chambers (Matsunami Glass, Tokyo, Japan) and cultured for assay-specific times (24–144 h). Cells were washed with PBS, fixed with 4% formaldehyde at room temperature for 10 min, and then permeabilized using 0.5% Triton X-100 containing PBS at room temperature for 5 min, or they were fixed and permeabilized with 100% ice-cold methanol at −30 °C for 15 min. Cells were then blocked with BlockAid Blocking Solution (Thermo Fisher Scientific) at room temperature for 1 h and reacted with primary antibodies (optimally diluted with BlockAid Blocking Solution) at 4 °C overnight. After cells were washed with PBS, fluorophore-labeled secondary antibody solution was added to the cells, and the mixture was incubated at room temperature for 1 h. The cells were then washed in PBS and counterstained with ProLong Glass Antifade Mountant with NucBlue Stain (Thermo Fisher Scientific) and/or Actin-stain 555 phalloidin (Cytoskeleton, CO, USA), according to the manufacturer’s protocol, and mounted with coverslips. All fluorescent images were acquired using a fluorescent microscope (BZ-X710; Keyence, Osaka, Japan), and the fluorescence area, co-localization, and numbers of nuclei were quantified by BZ-X Analyzer software (Keyence).

### Live cell imaging

LysoTracker (Thermo Fisher Scientific) and acridine orange (Sigma-Aldrich) were used to analyze the status of acidic organelles and lysosome membrane permeabilization in siRNA-treated cells. To monitor the localization of acidic organelles, treated cells were stained with 50 nM LysoTracker Deep Red containing growth medium at 37 °C for 1 h. Cells washed in PBS were cultured under normal conditions, and their fluorescence was detected by a BZ-X710 fluorescent microscope or by a flow cytometer (BD FACSVerse) using 640 nm excitation with 660/10 nm band-pass emission filters. Separately, to validate lysosome membrane permeabilization, cells were incubated with 5 μM acridine orange-containing growth medium at 37 °C for 15 min. After recovery by trypsinization, the acridine orange-derived green fluorescence of the cells was then analyzed by flow cytometry (BD FACSVerse) using 488 nm excitation with 527/32 nm band-pass emission filters.

### Mitochondrial membrane potential assay

The membrane potential of the mitochondria in the live cells was analyzed by a MitoProbe JC-1 assay (Thermo Fisher Scientific). Cells were cultured in 2 μM JC-1-containing growth medium for 30 min at 37 °C with 5% CO_2_, followed by recovery by trypsinization. After cells were washed with PBS they were analyzed by flow cytometry (BD FACSVerse) using 488 nm excitation with 527/32 nm and 586/42 nm band-pass emission filters.

### ROS detection

Cellular oxidative stresses were monitored using a cell-permeable fluorescent probe, CellROX Green reagent (Thermo Fisher Scientific). Briefly, cultured cells were incubated in 5 μM CellROX Green-containing growth medium for 1 or 2 h at 37 °C with 5% CO_2_. Following trypsinization, the recovered cells were stained with 7-AAD and analyzed using a BD FACSVerse flow cytometer. The intracellular ROS was calculated as the mean fluorescence intensity of the FL1 channel in the 7-AAD negative population.

### Lysosomal enzyme activity assay

The activities of endogenous lysosomal enzymes in live cells were detected with the Lysosomal Intracellular Activity Assay Kit (BioVision, Milpitas, CA, USA). In brief, cultured cells were reacted with Self-Quenched Substrate (BioVision) in 0.5% FBS-containing growth medium for 1 h at 37 °C with 5% CO_2_. Cells were then recovered by trypsinization and stained with 7-AAD. Using a flow cytometer (BD FACSVerse), the activities of viable cells (the 7-AAD^−^ fraction) were detected by the fluorescence intensity of the FL1 channel. Similarly, images of the fluorescence were also acquired using a fluorescent microscope (BZ-X710).

### Immunohistochemistry

Tissues were fixed in formalin and then embedded in paraffin. Tissue slices (4 μm thick) were deparaffinized in xylene and hydrated in ethanol. Antigens were retrieved by boiling in 10 mM sodium citrate buffer (pH 6.0) for 1 h. Endogenous peroxidase was inactivated in 3% H_2_O_2_-containing methanol at room temperature for 5 min. Slices were blocked with BlockAid Blocking Solution (Thermo Fisher Scientific) at room temperature for 30 min and were then reacted with primary antibodies (optimally diluted with BlockAid Blocking Solution) at 4 °C overnight. The slides were then washed in PBS and reacted with EnVision+ Dual Link System-HRP (Dako) at room temperature for 30 min. After the slides were washed with PBS, they were stained using DAB Substrate Buffer (Dako) and counterstained with Mayer’s hematoxylin. They were then dehydrated in ethanol and xylene and mounted with coverslips. All images were acquired using an upright microscope (BX63; Olympus, Tokyo, Japan).

### Statistical analysis

The required sample sizes were estimated on the basis of previous experience concerning similar experiments. Mean ± SD values were obtained from three independent experiments. GraphPad Prism 7 (GraphPad Software, CA, USA) was used for analysis. Statistical differences were determined using unpaired, two-tailed *t*-test with Welch’s correction, Dunnett’s or Tukey multiple comparisons test. Log-rank tests were applied for Kaplan–Meier survival curves. Paired *t-*test was applied in paired samples. The strength of association between two variables and the direction of the relationship was explored with non-parametric correlation coefficient analyses (spearman) (*p *<* 0.05, **p *<* 0.01, ***p *<* 0.001, ****p *<* 0.0001).

## Supplementary information

Supplementary tables

Supplementary Figure Legends

Supplementary Figure S1

Supplementary Figure S2

Supplementary Figure S3

Supplementary Figure S4

## Data Availability

The data that support the findings of this study are available from the corresponding author upon reasonable request.
