# Supplementary material for: Upregulation of IGF2R evades lysosomal dysfunction-induced apoptosis of cervical cancer cells via transport of cathepsins
Source: Cell Death Dis. 2019 Nov 20;10(12):876. doi: 10.1038/s41419-019-2117-9 (PMC6868013; doi:10.1038/s41419-019-2117-9)
Supplement: Supplementary file 1 — Supplementary tables [file 41419_2019_2117_MOESM1_ESM.docx]

**Supplementary table S1.** A list of genes whose expression is changed by IGF2R knockdown

| **Increased expression by IGF2R knockdown** | | | |
| --- | --- | --- | --- |
| **Gene Symbol** | **Probe Set ID** | **Fold change (vs. siCtrl)** | **Fold change (vs. Parent)** |
| *CREM* | 230511_at | 7.9 | 5.1 |
| *SNAP25* | 202508_s_at | 6.2 | 3.1 |
| *CREM* | 209967_s_at | 6.0 | 4.2 |
| *MSRB3* | 225790_at | 5.7 | 4.2 |
| *PLAUR* | 211924_s_at | 5.3 | 4.1 |
| *CRISPLD2* | 221541_at | 5.1 | 3.3 |
| *CREM* | 214508_x_at | 4.7 | 6.1 |
| *MSRB3* | 238583_at | 4.4 | 3.6 |
| *PLCL2* | 213309_at | 4.2 | 6.0 |
| *CREM* | 207630_s_at | 4.2 | 5.0 |
| *MSRB3* | 1554127_s_at | 4.2 | 3.3 |
| *TMEM87B* | 225411_at | 4.1 | 2.6 |
| *SFT2D3* | 226639_at | 4.1 | 2.1 |
| *CYFIP2* | 220999_s_at | 4.0 | 6.5 |
| *ALCAM* | 201951_at | 3.8 | 5.0 |
| *CLIC4* | 201559_s_at | 3.5 | 3.9 |
| *AGO4* | 227930_at | 3.4 | 3.0 |
| *LSM14A* | 212132_at | 3.2 | 2.9 |
| *MED28* | 224416_s_at | 3.1 | 2.4 |
| *ITPR1* | 203710_at | 3.0 | 6.7 |
| *ITPR1* | 216944_s_at | 2.9 | 6.0 |
| *CARS2* | 218153_at | 2.7 | 2.5 |
| *FECH* | 203116_s_at | 2.6 | 2.6 |
| *PTBP3* | 214698_at | 2.6 | 2.3 |
| *ACAD8* | 221669_s_at | 2.5 | 2.2 |
| *DCTD* | 201572_x_at | 2.5 | 2.5 |
| *CYP26B1* | 219825_at | 2.4 | 3.7 |
| *VPS8* | 209553_at | 2.4 | 2.5 |
| *BCAT2* | 203576_at | 2.3 | 2.6 |
| *NAMPT* | 1555167_s_at | 2.3 | 2.6 |
| *SGTA* | 201396_s_at | 2.2 | 2.8 |
| *SESN1* | 218346_s_at | 2.2 | 6.1 |
| *SHISA5* | 222986_s_at | 2.1 | 2.6 |

| **Decreased expression by IGF2R knockdown** | | | |
| --- | --- | --- | --- |
| **Gene Symbol** | **Probe Set ID** | **Fold change (vs. siCtrl)** | **Fold change (vs. Parent)** |
| *IGF2R* | 201393_s_at | 11.9 | 6.4 |
| *IGF2R* | 201392_s_at | 9.7 | 4.2 |
| *AP2B1* | 200615_s_at | 7.7 | 3.4 |
| *OSBPL10* | 219073_s_at | 5.3 | 2.9 |
| *ZCCHC17* | 223107_s_at | 5.0 | 4.4 |
| *ENOX2* | 204643_s_at | 4.8 | 5.7 |
| *SMIM20* | 225014_at | 4.7 | 2.9 |
| *ACOT13* | 204565_at | 4.7 | 2.6 |
| *ZCCHC17* | 223108_s_at | 4.5 | 3.1 |
| *STYX* | 228853_at | 4.5 | 2.6 |
| *GTF3C6* | 225083_at | 4.3 | 3.5 |
| *AP2B1* | 200612_s_at | 4.3 | 4.7 |
| *ENOX2* | 32042_at | 4.3 | 4.6 |
| *TMEM167A* | 224702_at | 4.2 | 2.7 |
| *CMAS* | 218111_s_at | 4.1 | 2.6 |
| *NMD3* | 231870_s_at | 4.1 | 3.6 |
| *DYNC1I2* | 211684_s_at | 4.1 | 3.2 |
| *ZNF593* | 204175_at | 3.9 | 3.8 |
| *PAAF1* | 218957_s_at | 3.9 | 3.6 |
| *UMAD1* | 226596_x_at | 3.9 | 3.1 |
| *MOSPD1* | 218853_s_at | 3.9 | 3.6 |
| *MGAT5* | 212098_at | 3.8 | 3.1 |
| *COG5* | 203629_s_at | 3.8 | 2.3 |
| *SKP1* | 200711_s_at | 3.7 | 4.5 |
| *C1D* | 200056_s_at | 3.7 | 3.7 |
| *NMD3* | 222497_x_at | 3.7 | 3.0 |
| *TAF13* | 227278_at | 3.7 | 2.8 |
| *BNIP3L* | 221479_s_at | 3.6 | 2.4 |
| *RER1* | 202297_s_at | 3.6 | 3.2 |
| *TOMM5* | 225036_at | 3.6 | 4.8 |
| *DNAJB11* | 223054_at | 3.6 | 2.4 |
| *RPS24* | 200061_s_at | 3.5 | 4.8 |
| *FYTTD1* | 224641_at | 3.4 | 2.7 |
| *SELT* | 217811_at | 3.4 | 2.4 |
| *FAM210B* | 224690_at | 3.3 | 5.1 |
| *SMIM19* | 225534_at | 3.3 | 4.5 |
| *ARL6IP4* | 218216_x_at | 3.3 | 3.3 |
| *SCML1* | 218793_s_at | 3.2 | 3.1 |
| *RRBP1* | 201204_s_at | 3.2 | 2.1 |
| *ARL6IP4* | 220597_s_at | 3.2 | 2.3 |
| *MAPRE1* | 200713_s_at | 3.1 | 4.5 |
| *BRCC3* | 229436_x_at | 3.1 | 4.4 |
| *CFDP1* | 203166_at | 3.1 | 3.9 |
| *VPS54* | 222627_at | 3.1 | 3.2 |
| *SLC2A1* | 201250_s_at | 3.0 | 3.1 |
| *ARNT* | 218221_at | 3.0 | 3.0 |
| *SKP1* | 207974_s_at | 3.0 | 2.5 |
| *PEX19* | 201707_at | 3.0 | 3.1 |
| *RABL6* | 223166_x_at | 3.0 | 3.5 |
| *DPCD* | 226009_at | 3.0 | 3.9 |
| *LRBA* | 212692_s_at | 3.0 | 3.4 |
| *DPCD* | 1553976_a_at | 2.9 | 2.9 |
| *TM2D2* | 224413_s_at | 2.9 | 4.6 |
| *SKAP2* | 225639_at | 2.9 | 2.5 |
| *NARS2* | 219217_at | 2.8 | 2.7 |
| *CCNY* | 224647_at | 2.8 | 2.2 |
| *BRCC3* | 221196_x_at | 2.8 | 3.4 |
| *NRM* | 225592_at | 2.7 | 4.9 |
| *OSGEP* | 209450_at | 2.7 | 2.8 |
| *ATL3* | 224893_at | 2.7 | 2.4 |
| *GPN3* | 218461_at | 2.7 | 6.2 |
| *PRUNE* | 209586_s_at | 2.7 | 2.7 |
| *CCDC28A* | 209479_at | 2.7 | 3.6 |
| *H1FX* | 204805_s_at | 2.6 | 3.6 |
| *KARS* | 200079_s_at | 2.6 | 2.1 |
| *WWOX* | 219077_s_at | 2.6 | 4.8 |
| *HTATSF1* | 202602_s_at | 2.5 | 2.2 |
| *ZNF106* | 217781_s_at | 2.5 | 3.5 |
| *KARS* | 200840_at | 2.5 | 3.4 |
| *ITGB1BP1* | 203336_s_at | 2.5 | 2.7 |
| *SELT* | 225561_at | 2.5 | 2.3 |
| *PARP2* | 204752_x_at | 2.5 | 4.3 |
| *TFDP1* | 212330_at | 2.4 | 2.6 |
| *ZNF106* | 222407_s_at | 2.4 | 2.8 |
| *TNS3* | 217853_at | 2.4 | 2.9 |
| *DNAJC3* | 225284_at | 2.4 | 2.6 |
| *AUP1* | 220525_s_at | 2.4 | 2.3 |
| *ZC3H7B* | 205877_s_at | 2.4 | 3.3 |
| *TSN* | 201515_s_at | 2.4 | 3.6 |
| *PARP2* | 215773_x_at | 2.4 | 4.5 |
| *MARCKS* | 201670_s_at | 2.4 | 2.3 |
| *RBBP8* | 203344_s_at | 2.3 | 4.6 |
| *TMEM41B* | 212622_at | 2.3 | 2.4 |
| *BRCC3* | 231913_s_at | 2.2 | 2.7 |
| *ALKBH5* | 234302_s_at | 2.2 | 2.2 |
| *SNAPC5* | 1554093_a_at | 2.2 | 2.3 |
| *CDC42SE2* | 224709_s_at | 2.2 | 5.3 |
| *HOXA3* | 235521_at | 2.1 | 2.1 |
| *SFR1* | 236027_at | 2.1 | 2.1 |
| *SKP1* | 200718_s_at | 2.1 | 2.3 |

**Supplementary table S2.** List of primers used in this study

| **Target gene** | **Forward primer (5′ to 3′)** | **Reverse primer (5′ to 3′)** |
| --- | --- | --- |
| *GAPDH* (qRT-PCR) | CCAGCAAGAGCACAAGAGGA | GCAACTGTGAGGAGGGGAGA |
| *IGF2R* (qRT-PCR) | GAAGTGCGTGGGTGTGATGT | CAGACGAGAATTGGGGCTTT |
| *RAB1B* (qRT-PCR) | CCTGGGGCATTTGAGTCTGT | GGGCATCTTGGAAAGTCACC |
| *RAB1A* (qRT-PCR) | CCAGTGCTAAGAATGCAACGA | CAGCAACCTCCACCTGACTG |
| *ATL3* (qRT-PCR) | GTCGTCTGGCAATGGATGAA | AAGCAGGTGACATCGGAGAAA |
| *ARCN1* (qRT-PCR) | TCTCGGTTCCATCTCATCCA | TCTCGGTTCCATCTCATCCA |
| *CMAS* (qRT-PCR) | ATTCAGGGGCCTTCCAGAG | TGATGGCGTCTCACAACAGA |
| *COG5* (qRT-PCR) | CAGACGAGAATTGGGGCTTT | CGAAGCAAATCACAGGCAAC |
| *DYNC1l2* (qRT-PCR) | TCCTCAGCCTCAAGGTGTGTT | GGGGAGTAGGGGAGAAGTCATT |
| *MGAT5* (qRT-PCR) | GGCGTTCAGATCCCTGCTAC | ATCCATGCGTCAGCCATTC |
| *RER1* (qRT-PCR) | TCGCTACCCACCAAACAGAA | ACCAGAATCGGCCAGAACAC |
| *SCAMP1* (qRT-PCR) | CCTTGCTACCTATTGCTGTTTTATG | TCCCCTTAAAATGCAAACCA |
| *SEC22B* (qRT-PCR) | TGTGTCCCGACCCTATTCCT | ATTGGCCACCATGATCCTCT |
| *VPS54* (qRT-PCR) | ATGGGGATTGGACTTTGTGG | TCATCCAAGGCAAAATCTGGT |
| *CTSB* (qRT-PCR) | TCAGCCACAAATCCAGCAAC | CACGGGGTAGCATCTTGGTT |
| *CTSD* (qRT-PCR) | GGAAGGAGTGAGAGGGGACA | CAAGGGGAGGACAACAGAGG |
| *CTSL* (qRT-PCR) | CAAAACCGTAAGCCCAGGAA | AAAAGCCCAACAAGAACCACA |
| *CTSZ* (qRT-PCR) | TAACGCTGGCTCCTGTGAAG | GGTCCTTGGCCTGGTAGTTG |
