# Supplementary material for: Upregulation of IGF2R evades lysosomal dysfunction-induced apoptosis of cervical cancer cells via transport of cathepsins
Source: Cell Death Dis. 2019 Nov 20;10(12):876. doi: 10.1038/s41419-019-2117-9 (PMC6868013; doi:10.1038/s41419-019-2117-9)
Supplement: Supplementary file 2 — Supplementary Figure Legends [file 41419_2019_2117_MOESM2_ESM.docx]

**Supplementary Figure Legends**

**Supplementary Fig. S1** Transcriptome analysis of IGF2R in patients with cervical cancer. **a** Somatic mutation landscape of selected genes in 306 cervical cancers (top panel). Each column represents an individual tumor. The bottom panel is a heatmap of mRNA expression with one-to-one correspondence with the landscape map. **b** Correlation between the mRNA expression of IGF2R and that of cognate receptors/ligands. Relationships in individual tumors (306 cases) are depicted in each dot. r_s_: Spearman’s correlation. **c** A scatter plot of IGF2R and its cognate receptors’ expression in 306 cervical tumors. Dots are three-toned, indicating expression levels (mean ± SD). These classifications are used in the survival analyses shown in Fig. 1f and Supplementary Fig. S1d. **d** Overall survival analyses of cervical cancer patient groups classified by mRNA expression of IGF1R (top panel) and INSR (bottom panel) **e** Contribution of IGF2R mRNA expression to overall survival in various cancers. The bar represents the survival rate at 5 years for each group. **p* < 0.05, ***p* < 0.01, ****p* < 0.001; log rank *p*-values. **f** Changes in IGF2R mRNA expression levels after radiotherapy (20 cases, left panel) and chemoradiotherapy (19 cases, right panel). The dots of individual patients are connected by lines, *p* values were calculated by paired t test.

**Supplementary Fig. S2** Phenotypic changes in cervical cancer cells after IGF2R knockdown. **a** Correlation between mRNA and protein expression of IGF2R (left panel) and IGF1R (right panel). a.u.: arbitrary unit. **b** Cell cycle analysis of cervical cancer cells at 144 h at IGF2R or IGF1R siRNA transfection. **c** Representative flow cytometry plots for apoptosis detection. Data are summarized in Fig. 2d. **d** Effect of IGF2R/IGF1R knockdown on the migratory abilities of cervical cancer cells. Representative images of the wound healing assay (right panel) and summary of wound recovered area (right panel) at 48 h after siRNA transfection. Scale bars represent 400 μm. **e** The role of IGF2R and IGF1R in the invasion of cervical cancer cells. Representative images of the Matrigel invasion assay (left panel) and invasion abilities (right panel) at 48 h after siRNA transfection. **f** Effect of IGF2R knockdown on IGF1R protein expression by using five siRNA (#2: HSS105256, #3: s7217, #4: s7218, #5: HSS105257, and #6: HSS105258). **g** Spheroid formation ability of BOKU at 6 days after IGF2R siRNA transfection. Representative images of colonies (left panel) and summary of colony sizes (right panel) are shown. a.u.: arbitrary unit. **h** Effect of IGF2R knockdown on cisplatin sensitivity. At 48 h after siRNA transfection, cells were further exposed to various concentrations of cisplatin for 96 h. The panel shows the dose-response curves for cisplatin (CDDP). Scale bars represent 400 μm. All the error bars represent the standard deviation of three independent experiments. **p* < 0.05, ***p* < 0.01, t test with Welch’s correction and Dunnett’s multiple comparisons test.

**Supplementary Fig. S3** Loss of IGF2R induces the accumulation of inactive lysosomes and autophagic dysfunction in cervical cancer cells. **a** Immunocytochemical staining of IGF2R (green) at 72 h after siRNA transfection. Nuclei and filamentous actin were counterstained with NucBlue (blue) and phalloidin (red), respectively. Scale bars represent 20 μm. **b** Time-lapse imaging of acidic organelles in IGF2R-knockdown cells. The fluorescence of LysoTracker was detected with the same exposure time. Scale bars represent 100 μm. **c** Distribution of lysosomes in IGF2R-knockdown cells. Immunocytochemical staining of a lysosome marker, LAMP1 (red), in IGF2R-knockdown cells (96 h). Scale bars represent 20 μm. **d** Influence of IGF2R knockdown on lysosome membrane permeability at 96 h after siRNA transfection. After staining with acridine orange, cells were analyzed for green fluorescence as an indicator of leakage of the dye from the lysosomes. Representative histograms of flow cytometry are shown. **e** Detection of lysosome activities in IGF2R-knockdown cells at 96 h after siRNA transfection. Green fluorescence indicates substrates that were cleaved by lysosomal enzymes. The nuclei of the cells were counterstained with NucBlue (blue). Scale bars represent 20 μm. **f** Mitochondrial membrane potential of IGF2R-knockdown cells. At 96 h after siRNA transfection, cells were analyzed by flow cytometry-based JC-1 assay (top panel). Total membrane potential was calculated from the ratio of JC-1 red fluorescence^+^ to JC-1 red fluorescence^-^ (bottom panel). All the error bars represent the standard deviation of three independent experiments. **p* < 0.05, ***p* < 0.01; t test with Welch’s correction.

**Supplementary Fig. S4** IGF2R has a dominant role in maintaining intracellular cathepsin B and L as an M6P receptor in cervical cancer cells. **a** mRNA expression profile of the cathepsin family in cervical cancer (306 cases). Boxes and bars represent the range from the first to third quartile and minimum to maximum, respectively. **b** Proteomic analysis of cathepsin expression in IGF2R-knockdown cells. Protein expression levels were calculated based on peak intensities obtained from mass spectrometry. **c** Contributions of the mRNA expression levels of cathepsins to the survival of patients with cervical cancer (291 cases). **p* < 0.05, log rank *p*-values. **d** Correlation analysis of the mRNA expression of IGF2R and cathepsins. Relationships in individual cervical tumors (306 cases) are depicted in each dot. r_s_: Spearman’s correlation. **e** Effect of IGF2R knockdown on the mRNA expression of cathepsins. In cells at 72 h after siRNA transection, mRNA expression of cathepsins was investigated by quantitative RT-PCR. **f** Influence of secreted factors from IGF2R-knockdown cells to cell growth. At 96 h after siRNA transfection, the cultured medium was recovered and added to the parental cells. Viable cells are detected 96 h after addition of the medium. **g** mRNA expression levels of M6PR in normal cervix (25 cases) and cervical cancer tissues (28 cases). Boxes and bars represent the range from the first to third quartile and minimum to maximum, respectively. **h** Comparison of overall survival in patients with cervical cancer (291 cases) with high and low M6PR mRNA expression levels. **i** Western blot analysis of polyubiquitinylated proteins and cathepsins in M6PR-knockdown cells. **j** Effect of M6PR knockdown on the survival of cervical cancer cells. **k** Detection of acidic organelles of cells with CK2 inhibitors. At 72 h after treatment with various concentrations of the inhibitors, cells were stained with LysoTracker and analyzed by flow cytometry. **l** Changes in the intracellular distributions of IGF2R caused by CK2 inhibitors. CX-4945 and CKII inhibitor VIII (CKIIi VIII) were used at a concentration of 5μM and 2μM, respectively. GM130 and Rab7 are markers for the Golgi apparatus and late endosomes, respectively. Scale bars represent 20 μm. All the error bars represent the standard deviation of three independent experiments. **p* < 0.05, ***p* < 0.01, ****p* < 0.001; t test with Welch’s correction and Dunnett’s multiple comparisons test.
