# Supplementary figures and images for: Upregulation of IGF2R evades lysosomal dysfunction-induced apoptosis of cervical cancer cells via transport of cathepsins
Source: Cell Death Dis. 2019 Nov 20;10(12):876. doi: 10.1038/s41419-019-2117-9 (PMC6868013; doi:10.1038/s41419-019-2117-9)

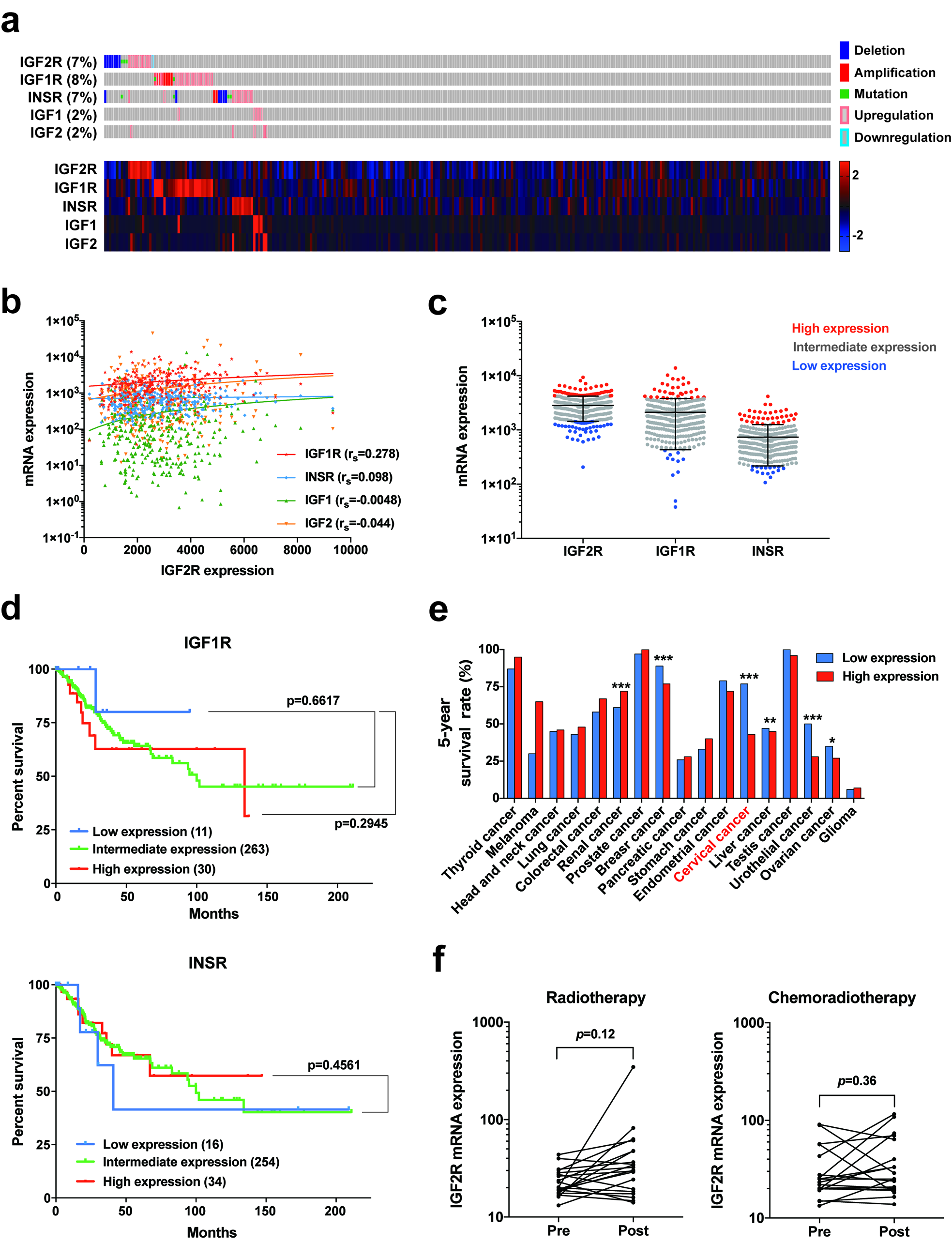

Supplement: Supplementary file 3 — Supplementary Figure S1 [file 41419_2019_2117_MOESM3_ESM.tif]

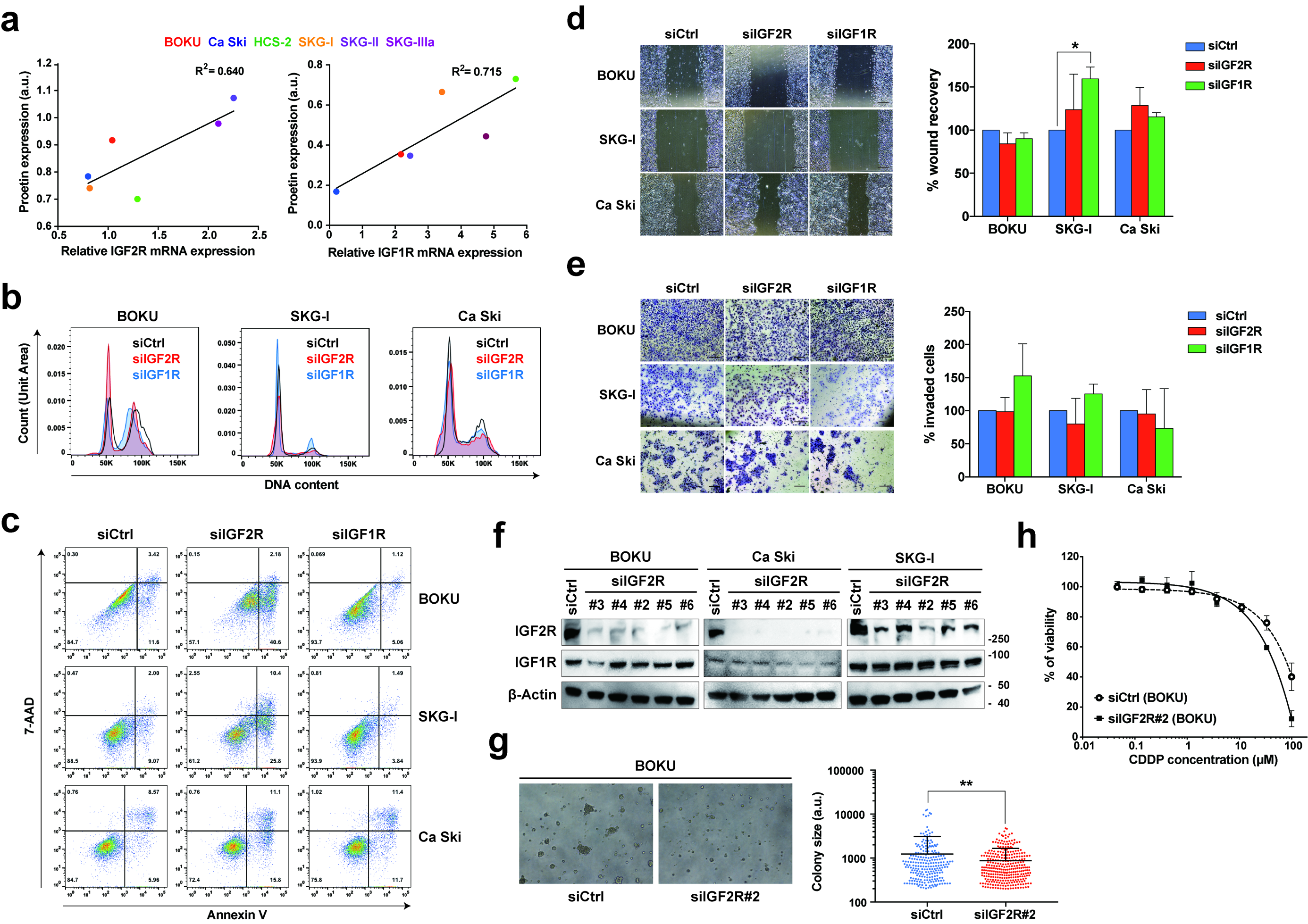

Supplement: Supplementary file 4 — Supplementary Figure S2 [file 41419_2019_2117_MOESM4_ESM.tif]

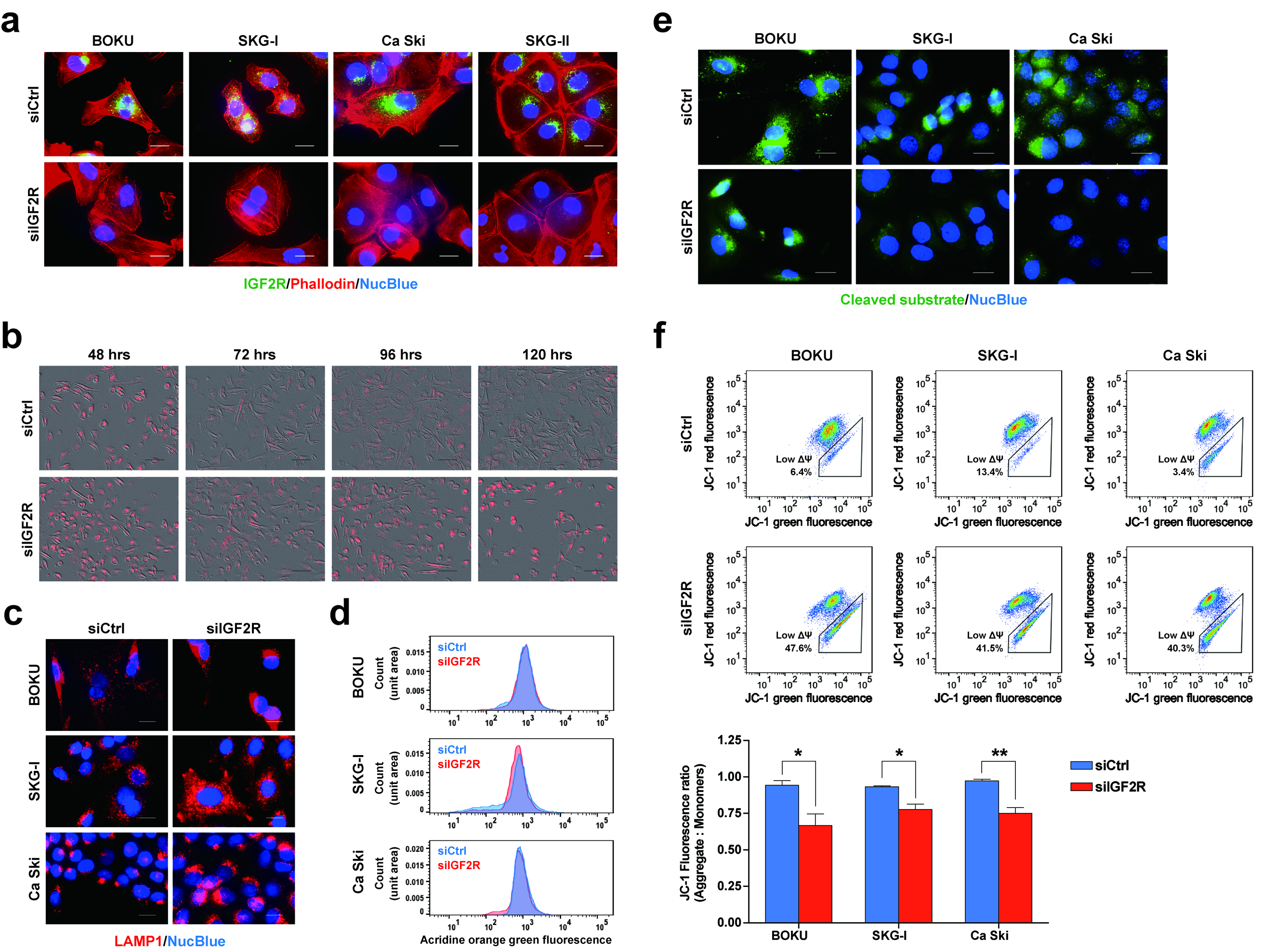

Supplement: Supplementary file 5 — Supplementary Figure S3 [file 41419_2019_2117_MOESM5_ESM.tif]

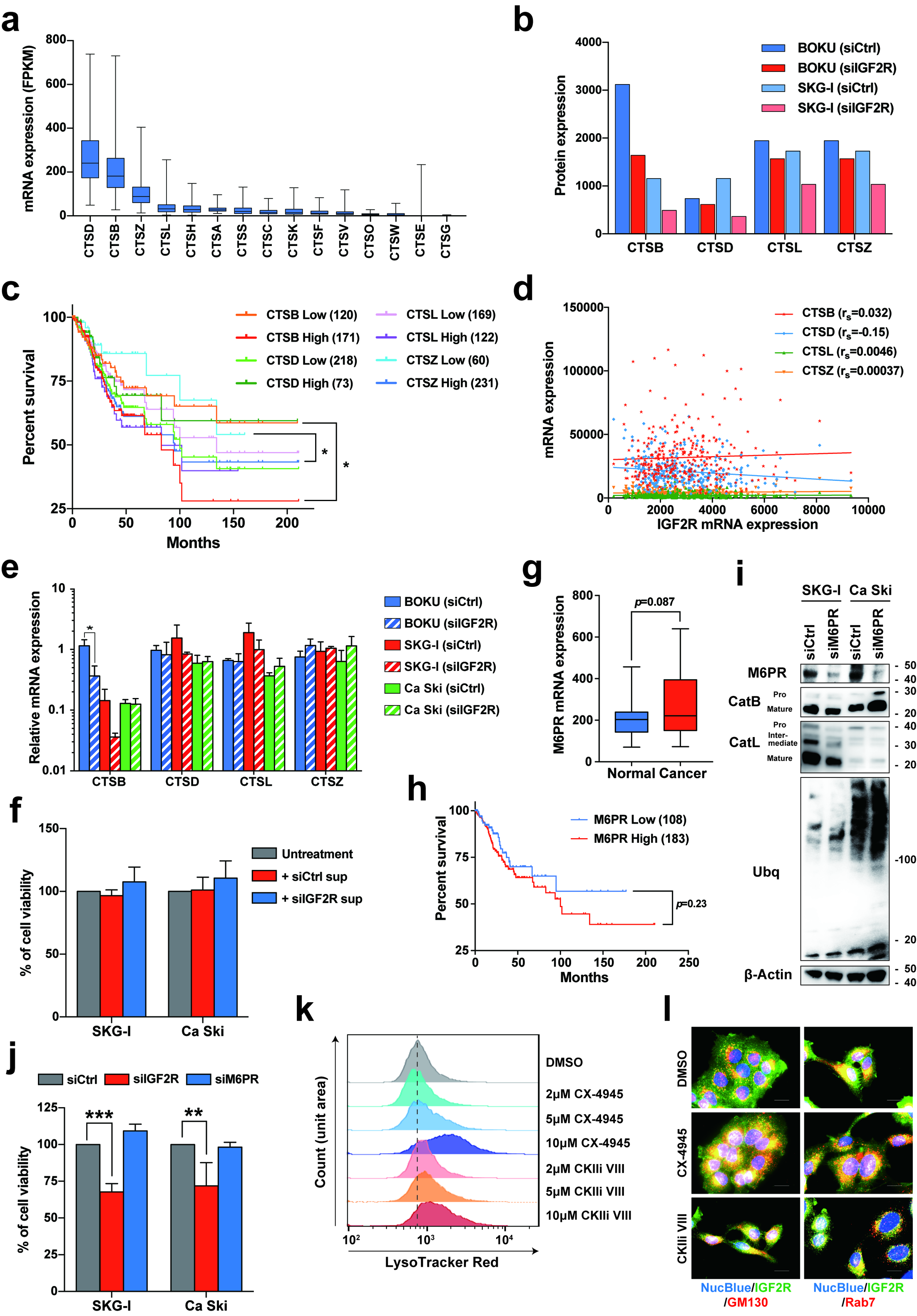

Supplement: Supplementary file 6 — Supplementary Figure S4 [file 41419_2019_2117_MOESM6_ESM.tif]
